# Halochromic Behavior and Anticancer Effect of New Synthetic Anthocyanidins Complexed with β-Cyclodextrin Derivatives

**DOI:** 10.3390/ijms23158103

**Published:** 2022-07-22

**Authors:** Iulia Păușescu, Izolda Kántor, György Babos, Zoltán May, Andrea Fodor-Kardos, Zsombor Miskolczy, László Biczók, Francisc Péter, Mihai Medeleanu, Tivadar Feczkó

**Affiliations:** 1 Faculty of Industrial Chemistry and Environmental Engineering, University Politehnica Timișoara, C. Telbisz 6, 300001 Timișoara, Romania; iulia.pausescu@upt.ro (I.P.); francisc.peter@upt.ro (F.P.); 2 Institute of Materials and Environmental Chemistry, Research Centre for Natural Sciences, Magyar Tudósok Körútja 2, H-1117 Budapest, Hungary; kantor.izolda@ttk.hu (I.K.); may.zoltan@ttk.hu (Z.M.); kardos@mukki.richem.hu (A.F.-K.); miskolczy.zsombor@ttk.hu (Z.M.); biczok.laszlo@ttk.hu (L.B.); 3 Faculty of Engineering, University of Pannonia, Egyetem u. 10, H-8200 Veszprém, Hungary; babos@mukki.richem.hu; 4 Research Institute for Renewable Energies, University Politehnica Timișoara, G. Muzicescu 138, 300501 Timișoara, Romania

**Keywords:** anthocyanidins, flavylium, cytostatic effect, inclusion complex, β-cyclodextrin derivatives

## Abstract

Anthocyanidins, the aglycons of anthocyanins, are known, beyond their function in plants, also as compounds with a wide range of biological and pharmacological activities, including cytostatic effect against various cancer cells. The nature and position of the substituents in the flavylium cation is essential for such biological properties, as well as the equilibrium between the multistate of the different chemical species that are generated by the flavylium cation, including quinoidal base, hemiketal, and *cis*- and *trans*-chalcones. In this work, eight new flavylium derivatives were synthesized, characterized for confirmation of the structure by FT-IR and 2D-NMR, and investigated in vitro as possible cytostatic compounds against HCT116 and HepG2 cancer cells. The most active two compounds were explored for their halochromic properties that can influence the biological activity and subjected to molecular encapsulation in β-cyclodextrin derivatives in order to increase their solubility in water and bioavailability. The anticancer effect was influenced by the position (6-, 7-, or 8-) of the methoxy group in the β-ring of the methoxy-4′-hydroxy-3′-methoxyflavylium cation, while the study of the halochromic properties revealed the important role played by the chalcone species of the pH-dependent multistate in both the uncomplexed and inclusion complex forms of these anthocyanidins.

## 1. Introduction

Anthocyanins are pigments with various colors that mostly present in plants. They have a basic flavonoid structure, carrying a positive charge at the oxygen atom. Their color changes from red to blue as a function of pH. Anthocyanins are mainly found in red-, purple-, and blue-colored flowers and fruits [1]. They are potential food additives for prevention of various diseases, exhibiting antidiabetic, anticancer, anti-inflammatory, and antimicrobial effects [2,3,4]. Anthocyanins are extremely sensitive to environmental conditions, such as temperature, pH, light, and oxygen. Due to their poor stability, encapsulation technologies were thoroughly investigated to improve the bioavailability of anthocyanins and to control their release in proper medium [5].

The chemotherapeutic and chemopreventive effect of many natural compounds is well known and has been used for developing new drugs with enhanced specificity and effectiveness in cancer therapy [6,7]. Thus, the research in this field evolved as a powerful approach for discovering biologically active compounds with unique structures and mechanisms of action [8]. Among a wide variety of natural compounds, anthocyanins and their derivatives were proved promising anticancer agents [9]. Mottaghipisheh et al. [10] gathered many preclinical and clinical studies about the role of anthocyanins in cancer treatment. Sorrenti et al. [11] confirmed that anthocyanins can induce apoptosis and suppression in prostate cancer cell proliferation. Medic et al. [12] published a literature review about the colorectal cancer prevention by anthocyanins, while Charapelli et al. demonstrated that anthocyanin-containing purple potato extracts suppress colon tumorigenesis [13]. A significant inhibition effect of cyanidin on renal carcinoma cells was reported by Liu et al. [14]. In addition, anthocyanins have a modulator role in expression of various tumor cell growth factors [15]. Anwar et al. [16] suggested that berry anthocyanins are possible chemotherapeutics for colon cancer. Asadi et al. [17] reported an anticancer effect of anthocyanins from sweet potato in mice, targeting intestinal adenomas.

The substitution pattern in the flavylium cationic structures of the natural anthocyanidins is essential for their properties, including color, stability, and biological activity, which are mainly controlled by the hydroxylation and O-methylation degree in the β-ring. The presence of vicinal hydroxy groups in the β-ring is considered detrimental for the stability, increasing the susceptibility for oxidation [18]. Considering the technological difficulties of anthocyanin separation in concentrated form, the synthesis of new anthocyanidins was considered an important tool to add novel functionalities and improve the stability and biological activity. Although the most common synthetic method involves the reaction of a substituted acetophenone with an aromatic aldehyde in acidic catalysis, other possible pathways, e.g., using natural benzopyrans as starting materials, have also been reported [19].

It is well known that the flavylium cation form of anthocyanidins is the stable species only in strongly acidic medium, while at increasing pH a quinoidal base as well as a hemiketal (by hydration) will be formed in equilibrium reactions. Subsequently, the central ring of the hemiketal can open to give *cis*-chalcone, which can further isomerize to *trans*-chalcone [20]. Chalcones represent an important class of natural products with cytotoxic, anticancer, anti-inflammatory, and antimicrobial activities, and the synthesis of chalcone derivatives was considered an important way towards new compounds with cytotoxic activities against human tumor cells [21]. Specifically, methoxylated chalcones have shown cytotoxic activities against different cancer cell lines [22]. Since chalcones are known to be phototransformed from *trans* into *cis* isomers in solution, it was assumed that the antitumorigenic activity of the *cis* analogues is higher compared to the *trans* isomers, although the evaluation of the individual efficacy of these compounds is difficult, considering that usually both isomers are present in the solution [23].

Cyclodextrins are cyclic oligosaccharides derived from starch, consisting of six or more D-glucopyranose units. The cone shaped cyclodextrins have versatile character because the walls are hydrophilic, while the central cavity has hydrophobic character. This feature allows cyclodextrins to incorporate nonpolar guest molecules inside the cavity, while the outer wall makes it more soluble or dispersible in water phase, forming inclusion complexes [24,25]. Inclusion complexes are produced mainly by hydrogen bonds, Van der Waals forces, or by hydrophobic interactions. This method is mostly used to cover compounds with small size and great volatility [26]. These complexes can improve the stability and solubility of the guest molecules but cyclodextrins also act as protectors against oxidative and heat damages. The use of cyclodextrins is widespread nowadays, especially in medicines and food products [27,28]. The hydrophilic derivatives of β-cyclodextrins, such as sulfobutylether-β-cyclodextrin (SBECD) and lipophilic ones, such as randomly-methylated-β-cyclodextrin (RAMEB), are preferentially used due to their non-toxic character and great binding capacity [29].

Ahmad et al. [30] reported an effectively controlled release of anthocyanins encapsulated in β-glucan and β-cyclodextrin particles. The coated particles were more resistant to gastric fluids and their bioavailability was increased. Furthermore, the results showed an increased phenolic content and antioxidant activity due to the protective effect of coating materials. Fernandes et al. [31] evidenced a thermal protection by β-cyclodextrin. According to the authors, anthocyanin-loaded β-cyclodextrin could be potential carrier and stabilizer for anthocyanins. Kalogeropoulos et al. [32] showed that the inclusion in β-cyclodextrin protected herb extract from thermal degradation. Vilanova and Solans [33] noted a significant increase in water solubility and stability of vitamin A palmitate, suggesting that inclusion complexes with β-cyclodextrins can be a solution for surfactant-free emulsions to increase the bioavailability of encapsulated agents. Fenyvesi et al. [29] prepared chrysin–cyclodextrin complexes and reported an improved solubility and permeability of chrysin complexed by β-cyclodextrin derivatives. Gago et al. demonstrated that for encapsulation of 4′-(2-hydroxyethoxy)-7-hydroxyflavylium, the association constants of β-cyclodextrin with the multistate species of this synthetic flavylium derivative follow the order *trans*-chalcone > quinoidal base > flavylium cation [20]. This conclusion was confirmed by the results of the same research group directed to the encapsulation of 3-methoxy-4′,7-dihydroxyflavylium. The formation of inclusion complexes with both the hemiketal and *trans*-chalcone forms was proven by using positive-induced circular dichroism. In the presence of β-cyclodextrin, the equilibrium was displaced toward the formation of the chalcone species, although β-cyclodextrin can interact with all species of the flavylium-based multistate in a dynamic equilibrium [34].

The main goal of our work was to synthesize new synthetic anthocyanidins with hydroxy and methoxy substituents in the flavylium cation; perform a comprehensive characterization of them, including a study of the halochromic properties that can provide useful information concerning the multistate species present in the system; and investigate the cytostatic effect on different cancer cell lines. The compounds with the highest anticancer efficiency were further subjected to molecular encapsulation in β-cyclodextrin derivatives to increase their water solubility, and the cytotoxicity of the resulted complexes was also evaluated. This is the first report of the anticancer effect of methoxy-4′-hydroxy-3′-methoxyflavylium inclusion complexes, emphasizing the influence of the methoxy substituent position.

## 2. Materials and Methods

### 2.1. Materials and Measurements

Sulfobutylether-β-cyclodextrin (SBECD) and randomly methylated β-cyclodextrin (RAMEB) were a kind gift provided by Cyclolab (Budapest, Hungary).

4′-Hydroxy-3′-methoxyacetophenone (97%), 2,3-dihydroxybenzaldehyde (97%), 2,4-dihydroxybenzaldehyde (98%), 2,5-dihydroxybenzaldehyde (98%), 2,3,4-trihydroxy- benzaldehyde (98%), 2-hydroxy-3-methoxybenzaldehyde (98%), 2-hydroxy-4-methoxy- benzaldehyde (98%), 2-hydroxy-5-methoxybenzaldehyde (98%), 2-hydroxy-4,6-dimethoxy-benzaldehyde (98%), boric acid (H_3_BO_3_, >99.5%), citric acid (>99.5%), and trisodium phosphate (Na_3_PO_4_, 96%) were purchased from Sigma Aldrich (Steinheim am Albuch, Germany). Sulfuric acid (H_2_SO_4_, 95–97%) and acetic acid (CH_3_COOH, 98%) were acquired from Merck KGaA (Darmstadt, Germany). Methanol (MeOH, >99%) and diethyl ether (>99%) were purchased from CHIMREACTIV SRL (Bucuresti, Romania).

The HepG2 and HCT116 cell lines were kind gift from the National Institute of Oncology (Budapest, Hungary).

The chemicals, reagents, and solvents were used without further purification.

FT-IR spectra were obtained on a Bruker Vertex 70 (Bruker Daltonik GmbH, Bremen, Germany) spectrometer equipped with a Platinium ATR, Bruker Diamond Type A225/Q in ATR (attenuated total reflectance) mode. The spectra of the samples were registered in the 4000 to 400 cm^−1^ spectral domain with a resolution of 4 cm^−1^ and 128 scans.

UV–VIS spectra were recorded on an Agilent Cary 60 spectrophotometer (Agilent Technologies, Waldbronn, Germany) at 25 °C.

NMR spectra were recorded on a Bruker Fourier 300 spectrometer (Bruker Daltonik GmbH, Germany) operating at 300 MHz (^1^H) and 75 MHz (^13^C) and on Bruker AVANCE III spectrometer (Bruker Daltonik GmbH, Germany) operating at 500.0 MHz (^1^H) and 125.0 MHz (^13^C) at 298 K. Chemical shifts δ are reported in ppm versus tetramethylsilane, TMS; coupling constants, *J*, are reported in Hz. The splitting patterns are abbreviated as: s (singlet), d (doublet), dd (doublet of doublets), t (triplet), and m (multiplet). The samples were dissolved in DMSO-*d_6_*. NMR assignments were based on 1D NMR spectra (^1^H, ^13^C, DEPT 135) and 2D NMR spectra (COSY, HQSC, HMBC) analysis.

The pH measurements of solutions were performed with a Mettler Toledo Seven Compact S210-K (Mettler Toledo, Columbus, OH, USA) at 25°C.

The melting points were determined on a Carl Zeiss melting point apparatus (Carl Zeiss, Oberkochen, Germany) and are uncorrected.

### 2.2. Methods

#### 2.2.1. Synthesis of the Bio-Inspired Anthocyanidins/Flavylium Compounds

The synthetic anthocyanidins were obtained by the acid-catalyzed condensation reaction of 4′-hydroxy-3′,5′-dimethoxyacetophenone (0.002 mol) and the appropriate 2-hydroxybenzaldehydes (0.002 mol) (2,3-dihydroxybenzaldehyde, 2,4-dihydroxy-benzaldehyde, 2,5-dihydroxybenzaldehyde, 2,3,4-trihydroxy-benzaldehyde, 2-hydroxy-3-methoxy-benzaldehyde, 2-hydroxy-4-methoxybenzaldehyde, 2-hydroxy-5-methoxy-benzaldehyde, 2-hydroxy-4,6-dimethoxybenzaldehyde). Following the method already reported in the literature [35], the reagents were dissolved in 12 mL acetic acid, and then 3 mL sulfuric acid 97% was added. The reaction mixtures were stirred magnetically at room temperature for 24 h. During this period, the formation of solids was observed. To improve the precipitation process, diethyl ether was added. The solids were separated by filtration, washed with diethyl ether, and dried at room temperature, yielding the anthocyanidins in hydrogensulfate salt form. The purity of all obtained compounds was calculated on the basis of NMR analysis. Because these values were in all cases higher than 94%, further purification was not necessary.

*6-Hydroxy-4′-hydroxy-3′-methoxyflavylium hydrogensulfate (1)*

410 mg of red precipitate (98.6% purity), η = 56.1%, m.p. 204–206 °C

FT-IR (ATR) cm^−1^: 3425; 3082; 2980; 2889; 1718; 1618; 1595; 1549; 1500; 1472; 1385; 1296; 1149; 1115; 1016; 932; 856; 812; 744; 648; 567; 473; 430.

^1^H-NMR (500 MHz, DMSO-*d_6_*, δ ppm): 9.23 (d, *J* = 9.2 Hz, 1H), 8.81 (d, *J* = 9.2 Hz, 1H), 8.28 (d, *J* = 9.3 Hz, 1H), 8.24 (d, *J* = 10.8 Hz, 1H), 8.03 (d, *J* = 2.1 Hz, 1H), 7.73 (dd, *J* = 9.2, 2.9 Hz, 1H), 7.50 (d, *J* = 2.9 Hz, 1H), 7.14 (d, *J* = 8.6 Hz, 1H), 3.99 (s, 3H).

^13^C-NMR (125 MHz, DMSO-*d_6_*, δ ppm): 171.8; 157.6; 157.2; 152.2; 149.6; 148.9; 128.2; 127.3; 124.9; 120.4; 119.7; 117.4; 117.0; 112.5; 111.0; 56.2.

*7-Hydroxy-4′-hydroxy-3′-methoxyflavylium hydrogensulfate (2)*

277 mg of burgundy precipitate (99.5% purity), η = 37.9%, m.p. 212–214 °C

FT-IR (ATR) cm^−1^: 3477; 3093; 2980; 2887; 1749; 1628; 1589; 1562; 1539; 1500; 1475; 1392; 1325; 1300; 1163; 1134; 1018; 962; 932; 847; 824; 744; 689; 658; 563; 440.

^1^H-NMR (300 MHz, DMSO-*d_6_*, δ ppm): 9.20 (d, *J* = 8.8 Hz, 1H), 8.56 (d, *J* = 8.8 Hz, 1H), 8.18 (t, *J* = 10.3 Hz, 2H), 7.97 (s, 1H), 7.60 (s, 1H), 7.42 (dd, *J* = 8.9, 2.2 Hz, 1H), 7.12 (d, *J* = 8.6 Hz, 1H), 3.98 (s, 3H).

^13^C-NMR (75 MHz, DMSO-*d_6_*, δ ppm): 171.1; 168.0; 158.2; 156.3; 153.0; 148.9; 132.7; 126.2; 120.9; 119.3; 118.3; 117.0; 112.9; 112.4; 102.8; 56.3.

*8-Hydroxy-4′-hydroxy-3′-methoxyflavylium hydrogensulfate (3)*

312 mg of burgundy precipitate (95.9% purity), η = 42.6%, m.p. 233–235 °C

FT-IR (ATR) cm^−1^: 3053; 1554; 1512; 1468; 1439; 1352; 1331; 1277; 1252; 1186; 1149; 1082; 1024; 918; 864; 839; 791; 741; 660; 567; 540; 480; 438.

^1^H-NMR (500 MHz, DMSO-*d_6_*, δ ppm): 9.30 (d, *J* = 9.2 Hz, 1H), 8.86 (d, *J* = 9.2 Hz, 1H), 8.27 (dd, *J* = 8.7, 2.2 Hz, 1H), 8.04 (s, 1H), 7.75–7.61 (m, 3H), 7.19 (d, *J* = 8.7 Hz, 1H), 3.98 (s, 3H).

^13^C-NMR (125 MHz, DMSO-*d_6_*, δ ppm): 172.9; 158.1; 153.4; 148.9; 146.5; 144.4; 129.4; 127.9; 124.5; 122.4; 119.7; 119.5; 117.5; 117.4; 113.0; 56.1.

*7,8-Dihydroxy-4′-hydroxy-3′-methoxyflavylium hydrogensulfate (4)*

487 mg of dark orange precipitate (95.7% purity), η = 63.7%, m.p. 194–196 °C

FT-IR (ATR) cm^−1^: 3157; 2980; 2889; 1747; 1632; 1597; 1560; 1516; 1479; 1448; 1396; 1323; 1302; 1250; 1186; 1136; 1020; 966; 870; 825; 748; 710; 710; 654; 559; 434.

^1^H-NMR (500 MHz, DMSO-*d_6_*, δ ppm): 9.18 (d, *J* = 8.9 Hz, 1H), 8.53 (d, *J* = 8.8 Hz, 1H), 8.20 (dd, *J* = 8.6, 2.2 Hz, 1H), 8.02 (s, 1H), 7.75 (d, *J* = 8.8 Hz, 1H), 7.47 (d, *J* = 8.8 Hz, 1H), 7.15 (d, *J* = 8.6 Hz, 1H), 3.97 (s, 3H).

^13^C-NMR (125 MHz, DMSO-*d_6_*, δ ppm): 170.9; 156.2; 156.1; 153.4; 148.6; 146.0; 132.8; 126.2; 122.4; 120.0; 119.7; 118.8; 116.9; 112.5; 112.4; 56.1.

*6-Methoxy-4′-hydroxy-3′-methoxyflavylium hydrogensulfate* *(5)*

373 mg of bright red precipitate (94.6% purity), η = 49%, m.p. 192–194 °C

FT-IR (ATR) cm^−1^: 3292; 3101; 2980; 2889; 1701; 1616; 1583; 1545; 1502; 1468; 1379; 1340; 1269; 1215; 1175; 1111; 1057; 1011; 947; 930; 874; 825; 735; 646; 557; 455.

^1^H-NMR (500 MHz, DMSO-*d_6_*, δ ppm): 9.24 (d, *J* = 9.2 Hz, 1H), 8.85 (d, *J* = 9.2 Hz, 1H), 8.34 (d, *J* = 9.3 Hz, 1H), 8.25 (d, *J* = 8.6 Hz, 1H), 8.02 (s, 1H), 7.86 (dd, *J* = 9.3, 3.0 Hz, 1H), 7.77 (d, *J* = 2.9 Hz, 1H), 7.15 (d, *J* = 8.6 Hz, 1H), 3.99 (s, 3H), 3.97 (s, 3H).

^13^C-NMR (125 MHz, DMSO-*d_6_*, δ ppm): 172.1; 158.7; 157.6; 152.2; 150.5; 149.0; 128.1; 127.6; 124.7; 120.4; 119.6; 117.7; 117.1; 112.6; 108.8; 56.3; 56.2.

*7-Methoxy-4′-hydroxy-3′-methoxyflavylium hydrogensulfate (6)*

468 mg of burgundy precipitate (97.1% purity), η = 61.6%, m.p. 252–254 °C

FT-IR (ATR) cm^−1^: 3088; 2980; 1632; 1576; 1529; 1500; 1468; 1373; 1342; 1300; 1219; 1130; 1057; 1016; 962; 932; 851; 752; 692; 652; 579; 525; 469; 413.

^1^H-NMR (500 MHz, DMSO-*d_6_*, δ ppm): 9.24 (d, *J* = 8.9 Hz, 1H), 8.65 (d, *J* = 8.9 Hz, 1H), 8.22 (t, *J* = 10.1 Hz, 2H), 8.00 (s, 2H), 7.52 (dd, *J* = 9.0, 2.4 Hz, 1H), 7.12 (d, *J* = 8.6 Hz, 1H), 4.11 (s, 3H), 4.00 (s, 3H).

^13^C-NMR (125 MHz, DMSO-*d_6_*, δ ppm): 171.6; 167.7; 157.9; 156.8; 152.9; 148.9; 131.6; 126.7; 120.4; 119.6; 118.8; 116.9; 114.0; 112.3; 100.8; 57.2; 56.2.

*8-Methoxy-4′-hydroxy-3′-methoxyflavylium hydrogensulfate (7)*

427 mg of red precipitate (94.1% purity), η = 56.2%, m.p. 256–259 °C

FT-IR (ATR) cm^−1^: 3078; 2943; 1589; 1554; 1512; 1456; 1439; 1418; 1319; 1288; 1219; 1138; 1101; 1011; 916; 872; 795; 748; 708; 656; 569; 527; 486; 444; 407.

^1^H-NMR (500 MHz, DMSO-*d_6_*, δ ppm): 9.33 (d, *J* = 9.2 Hz, 1H), 8.91 (d, *J* = 9.2 Hz, 1H), 8.19 (d, *J* = 8.6 Hz, 1H), 7.96 (s, 1H), 7.85–7.78 (m, 3H), 7.19 (d, *J* = 8.6 Hz, 1H), 4.15 (s, 3H), 3.97 (s, 3H).

^13^C-NMR (125 MHz, DMSO-*d_6_*, δ ppm): 172.8; 158.5; 153.2; 149.0; 147.7; 144.7; 132.7; 129.3; 127.8; 124.2; 120.5; 118.3; 117.9; 117.6; 112.8; 57.0; 56.0.

*5,7-Dimethoxy-4′-hydroxy-3′-methoxyflavylium hydrogensulfate (8)*

391 mg of red precipitate (94.4% purity), η = 47.6%, m.p. > 350 °C

FT-IR (ATR) cm^−1^: 3088; 2943; 2885; 1605; 1500; 1464; 1423; 1329; 1207; 1155; 1043; 822; 577.

^1^H-NMR (500 MHz, DMSO-*d_6_*, δ ppm): 9.08 (d, *J* = 8.9 Hz, 1H), 8.45 (d, *J* = 8.9 Hz, 1H), 8.12 (d, *J* = 8.5 Hz, 1H), 7.94 (s, 1H), 7.55 (s, 1H), 7.09 (d, *J* = 8.6 Hz, 1H), 6.95 (s, 1H), 4.09 (s, 3H), 4.07 (s, 3H), 3.97 (s, 3H).

^13^C-NMR (125 MHz, DMSO-*d_6_*, δ ppm): 171.1; 169.7; 158.0; 157.8; 156.3; 148.8; 147.5; 126.2; 119.5; 116.8; 112.2; 112.1; 112.0; 99.7; 93.8; 57.4; 57.3; 56.2.

#### 2.2.2. Spectroscopic Study of Halochromic Properties

For the halochromic properties via spectroscopy study, the buffer solutions (pH values ranging from 2 to 12) were prepared following a previously described procedure from boric acid 0.2 M, citric acid 0.005 M, and trisodium phosphate 0.1 M aqueous solutions [36]. UV–VIS spectra of the synthetic anthocyanidins solutions (compounds **5** and **7,** 7 × 10^−5^ M in methanol: water 1:14) at different pH values were measured in time.

#### 2.2.3. In Vitro Cytotoxicity Study

The HepG2 (hepatocellular carcinoma cells) was grown in Dulbecco’s modified Eagle’s medium (pH 7.4) supplemented with 10% fetal calf serum (FCS), and HCT116 (colon cells) were cultivated in RPMI-1640 medium (pH 7.4). The cells were cultured and incubated throughout the whole study at 37 °C in a humidified atmosphere containing 5% carbon dioxide. The cells were trypsinised, resuspended, and precultured before use.

In vitro cytotoxicity study of flavylium compounds was performed using 3-(4,5-dimethylthiazol-2-yl)-2,5-diphenyltetrazolium bromide (MTT) assay in two different cancer cell lines, i.e., HepG2 and HCT116. Cells were seeded (10000 cells/well) in 96-well plates. After 24 h pre-incubation, the growth media were replaced with 200 μL of fresh RPMI-1640 or DMEM media containing 10% FCS and MilliQ water solution of the compounds or their complexes with cyclodextrin. Concentrations of 26 μM, 66 μM, and 132 μM flavylium were calculated for the flavylium hydrogensulfate derivatives, with one methoxy substituent in the benzopyrylium cation applied to HepG2 and HCT116 cell lines. Since preliminary studies showed that RAMEB enhanced the water solubility of **7** from 2.3 mg/mL to 9.4 mg/mL and SBECD increased that of **5** from 6.3 mg/mL to 9.2 mg/mL, the cytotoxicity of these complexes was also studied using 10 μg, 5 μg, and 2 μg flavylium plus 10-fold higher mass of cyclodextrin per well in HepG2 cells. A total of 100 μg RAMEB or 100 μg SBECD per well was used as positive control.

After 48 h incubation, the media were discarded, and 20 μL/well MTT solution (5 mg MTT/mL) plus 0.2 mL/well culture media were pipetted, and the incubation was followed for 2 h. The supernatant was removed, and then the MTT lysis solution (DMSO, 1% acetic acid, 10% SDS) was added into each well to dissolve the cells containing MTT formazan crystals. The absorbance was determined at 570 nm by a Thermo Scientific Multiscan Sky plate reader (Bio-Science Kft., Budapest, Hungary). The viability of treated cells was related to the untreated cells (negative control). The data were presented as mean and standard deviation with 8 replicates.

#### 2.2.4. Investigation of Molecular Encapsulation by Cyclodextrins

Molecular encapsulation of the two flavylium compounds (**5**, **7**), which were effective against the two investigated cancer cell lines (HepG2 and HCT116), was achieved by cyclodextrins. Compounds **5** and **7** were dissolved in the aqueous solution of 20.84 mg/mL SBECD and 11.43 mg/mL RAMEB, respectively, in order to investigate their spectroscopic change in the presence of cyclodextrins. Absorption spectra of flavylium-loaded cyclodextrin solutions were recorded by a Cary 60 UV–VIS spectrophotometer (Agilent Technologies, Santa Clara, CA, USA). Dilute dye solutions with absorbance below 0.25 in the visible spectral range at 1 cm optical path were employed to avoid the formation of aggregates.

#### 2.2.5. Data Processing

The data were classified into two or three groups, according to the different concentration levels of flavilium compounds (132 μM, 66 μM, and 26 μM). Outliers were visualized by box and whiskers plots and were excluded from the statistical analysis. For the analysis of variances, *t*-test and one-way ANOVA were used. For testing the normality of distributions, the Shapiro–Wilk test was used. The homogeneity of variances was tested by F-test and Bartlett test at a significance level of 95%. In some cases, when the distributions of data were not normal or the variances were not equal, the proper non-parametric tests (Welch’s *t*-test, Mann–Whitney U-test, Kruskal–Wallis test) were used to compare the mean viability between the groups. When the one-way ANOVA or Kruskal–Wallis test showed significant differences in the means, the proper post hoc test (Tukey HSD or Kruskal–Nemenyi) was used to identify the actual differences between the pairs. All the statistics were carried out by using R software, version 3.4.0.

## 3. Results and Discussion

### 3.1. Synthesis and Characterization of the Anthocyanidins

**Figure 1:**
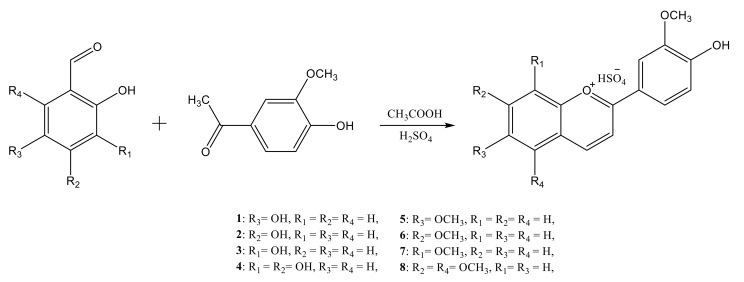
Reaction scheme for the synthesis of the synthetic anthocyanidins.

Eight new anthocyanidins were synthesized through acid-catalyzed (mixture of acetic acid and sulfuric acid 4:1, *v*/*v*) condensation reactions between 4′-hydroxy-3′,5′-dimethoxyacetophenone and substituted salicylaldehydes (Figure 1).

All synthesized compounds were characterized by FT-IR, UV–VIS, and NMR spectroscopy, with the structural analysis results demonstrating the formation of the targeted products.

The complete characterization is discussed only for 6-methoxy-4′-hydroxy-3′-methoxyflavylium hydrogensulfate (compound **5**) since there were only minor structural differences in the series of the synthetic anthocyanidins.

The FT-IR analysis confirmed the presence of the main functional groups and structural elements in compound **5**, evidenced by the following characteristic absorption bands: 3292 cm^−1^ (ν_OH_), 3101 cm^−1^ (ν_CarH_), 2980 cm^−1^ ($\nu_{CH3}^{as}$), 2889 cm^−1^ ($\nu_{CH3}^{s}$), 1701 cm^−1^ (ν_C=O+_), 1616, 1583 cm^−1^ (ν_sk,ar”1600”_), 1502, 1468 cm^−1^ (ν_sk,ar”1500”_), 1340 cm^−1^ ($\nu_{Car-O}^{as}$), 1111 cm^−1^ ($\nu_{COC}^{s}$), 1057 cm^−1^ ($\nu_{Car-O}^{s}$), 874, 825 cm^−1^ (1,2,4-trisubstituted benzene ring). The FT-IR spectrum is presented in the Supplementary Materials (Figure S1).

The exact structure of compound **5** was established by NMR analysis. In the ^1^H-NMR spectrum, the signals at 3.99 and 3.97 ppm, which are specific to aliphatic protons, were attributed to the protons of the methoxy groups (-OCH_3_). The signals corresponding to the aromatic protons were found between 7.15 and 9.24 ppm. The ^13^C-NMR spectrum revealed signals at 56.3 and 56.2 ppm corresponding to the carbon atoms involved in the methoxy groups; the aromatic carbon atoms were attributed to signals between 108.8 and 172.1 ppm. The most deshielded carbons were those linked to O atoms (172.1 ppm C=O^+^, 158.7 ppm C-OCH_3_, 157.6 ppm C-OH, 150.5 ppm C-O^+^, 149.0 ppm C-OCH_3_).

**Figure 2:**
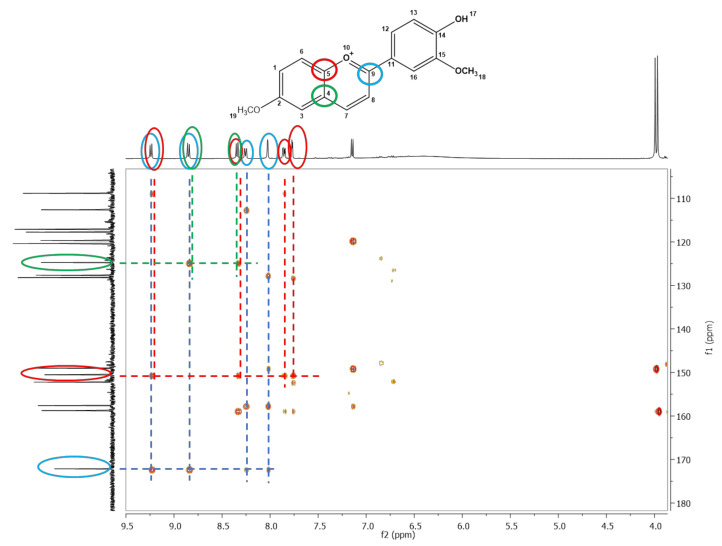
The ^1^H-^13^C-HMBC spectrum of 6-methoxy-4′-hydroxy-3′-methoxyflavylium hydrogensulfate (compound **5**)-the remote couplings between carbon atoms and protons are depicted by different coloured circles, green–C4 with H6 and H8, red–C5 with H3, H6 and H7, blue–C9 with H7, H8, H12 and H16.

The formation of compound **5** was further proven by the remote couplings between carbon atoms and protons, shown in the two-dimensional HMBC spectrum (Figure 2). The signal corresponding to the C9 (172.1 ppm) carbon atom coupled remotely (over two bonds) with protons H7 (9.22 ppm), H8 (8.85 ppm), H12 (8.25 ppm), and H16 (8.02 ppm). The coupling between the signal corresponding to the C5 (150.5 ppm) carbon atom with four proton signals: H7 (8.85 ppm), H6 (8.34 ppm), H1 (7.86 ppm), and H3 (7.77 ppm) can also be observed. Moreover, the remote coupling over two bonds of C4 (124.7 ppm) carbon atom with H6 (8.34 ppm) and H8 (8.85 ppm) protons confirmed the formation of compound **5**.

For all the other synthesized flavylium dyes, the characterization details are presented in the Supplementary Materials.

The FT-IR analysis demonstrated the presence of the main functional groups and structural elements of the synthesized compounds. All FT-IR spectra are presented in the Supplementary Materials (Figures S2–S8). The NMR analysis of 1D NMR (^1^H, ^13^C, DEPT 135) and 2D NMR (COSY, HQSC, HMBC) spectra confirmed the exact structures and purities of the synthesized anthocyanidins through the identification of the signals corresponding to the aliphatic and aromatic protons and carbon atoms in the ^1^H-NMR and ^13^C-NMR spectra, which are also presented in the Supplementary Materials (Figures S9–S24).

### 3.2. Cytotoxicity of the New Flavylium Compounds

**Figure 3:**
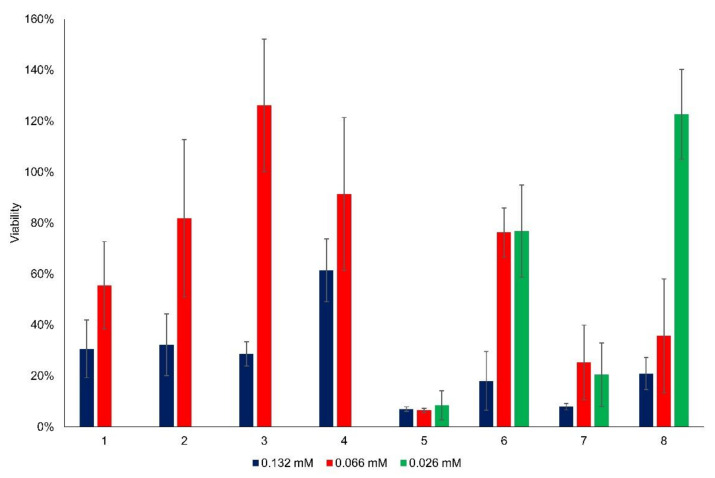
Viability (%) of HCT116 cells as a function of flavylium concentration.

**Figure 4:**
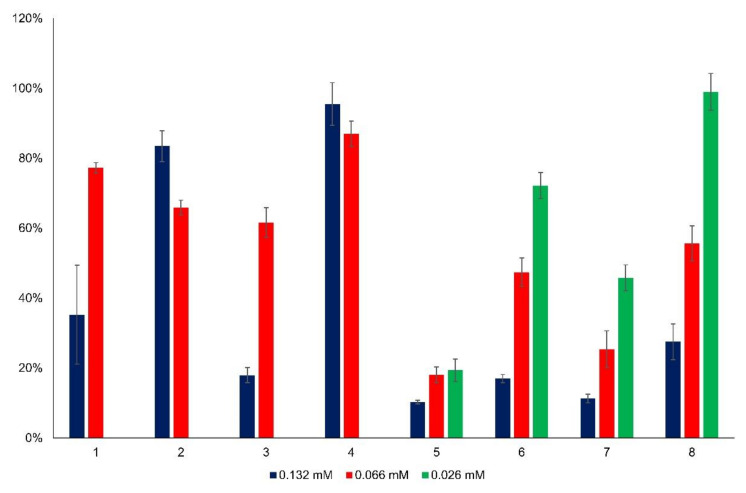
Viability (%) of HepG2 cells as a function of flavylium concentration.

Cytotoxicity of the changing concentration of flavylium compounds was investigated in vitro in HCT116 and HepG2 cancer cell lines. Figure 3 and Figure 4 summarize the viability data, where the results indicating effective cell inhibition are marked with bold letters. At the highest active agent concentration of 132 μM, most of the flavyliums inhibited the cancer cell growth effectively (Figure 3 and Figure 4). Exclusively, compound **4** showed inhibition in neither of the tumorous cells, and compound **2** had a cytotoxic effect in HCT116 but not in HepG2 cells. Using half concentration 66 μM, compounds **5**, **7**, and **8** inhibited the cell growth in both cell lines, and **6** exerted a substantial effect in HepG2. At the lowest concentration 26 μM, **5** and **7** were found to be effective cytotoxic agents in both cell lines.

Essentially, there are two main groups among the synthesized new flavylium compounds, (i) substituted by one or two hydroxy groups in the 2-(4′-hydroxy-3′-methoxy)phenyl-1-benzopyrylium basic structure, and (ii) substituted by one or two methoxy groups in the 2-(4′-hydroxy-3′-methoxy)phenyl- 1-benzopyrylium basic structure. Obviously, the anticancer effect is linked with the presence and position of these functional groups in the molecule, the cell growth inhibition being higher when the substituent was a methoxy group. It can be also easily observed that neither two methoxy substituents (compound **8**) nor two hydroxy substituents (compound **4**) in the benzopyrylium cation were beneficial for the anticancer effect, no matter if HCT116 cells or HepG2 cells were investigated. As concerns the position of the monosubstitution, positions 6 (in compound **5**) and 8 (in compound **7**) both favored the inhibition of cancer cell growth compared to position 7 (in compound **6**) when the substituent was methoxy, particularly at lower flavylium derivative concentrations, 66 μM and 26 μM, respectively. The best results were obtained with compound **5**, more than 90% inhibition for HCT 116 cells and more than 80% inhibition for HepG2 cells, both at 26 μM concentration. In the case of hydroxy substituents, the same positional effect was observed only at higher flavylium derivative concentration 132 μM, principally on the hepatic carcinoma HepG2 cells. In the case of flavylium compounds **1**–**4**, the statistical comparison of the mean viability between the two higher concentration groups (132 μM and 66 μM) in both cell lines showed significant differences in each case (Table S1). The higher concentration of flavylium derivatives was more effective than the lower one. Table S2 contains the statistical comparison of flavylium compounds **5**–**8** for each concentration group in both cell lines. In the case of compound **5** on HCT116 cells, there was no significant difference between the added amounts, and each concentration level showed the same effective cytotoxicity. However, in HepG2 cells, the cytotoxicity differed significantly in relation of 132 μM—66 μM and 132 μM—26 μM concentrations. In the case of compounds **6** and **7**, significant differences were observed between 132 μM—66 μM and 132 μM—26 μM concentrations in HCT116 and between 66 μM—26 μM and 132 μM—26 μM concentrations in HepG2 cells. In the case of 8 on HepG2 cells, a significantly decreasing cytotoxicity effect was revealed by decreasing the added concentrations.

The cytotoxic effect of anthocyanins, chalcones and their tautomerization product is known. Bandgar et al. demonstrated the anticancer effect of 2,4-dimethoxy- and 2,4,5-trimethoxy-chalcone derivatives on human cancer cells [22], but such an influence of the substituent position on the anticancer effect of synthetic anthocyanidins has not yet been reported. On the basis of these results, compounds **5** and **7**, both showing a very consistent anticancer effect even at low concentrations, were selected for the forthcoming investigations concerning the halochromic properties and formation of inclusion complexes with cyclodextrin derivatives.

### 3.3. Study of Halochromic Properties

**Figure 5:**
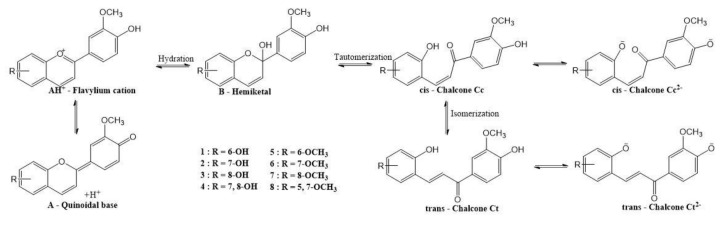
The network of chemical reactions of the synthetic anthocyanidins upon pH change.

It is generally accepted that flavylium salts (AH^+^) can be converted into quinoidal base (A) by fast deprotonation. A parallel hemiketal (B) formation also occurs via nucleophilic addition of water and proton loss in a concerted process [37,38]. The latter form tautomerizes to *cis*-chalcone (Cc), which isomerizes to *trans*-chalcone (Ct) [39,40]. All these species are produced in reversible reactions. Figure 5 presents the proposed pH-dependent equilibria of the synthesized anthocyanidins according to literature data [39].

**Figure 6:**
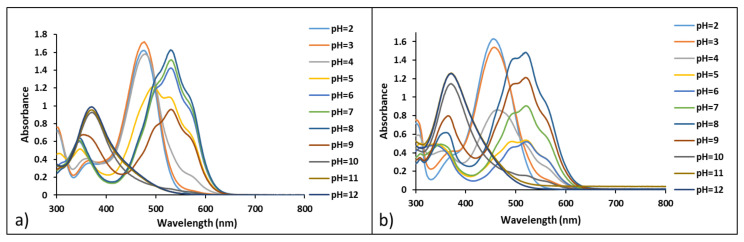
UV–VIS spectra of compounds **5** (**a**) and **7** (**b**) in different pH buffer solutions (7 × 10^−5^ M in methanol/water 1:14), 30 min after preparation.

The halochromic properties of compounds **5** and **7** were investigated by performing a UV–VIS spectroscopy study. The color variations of solutions at pH ranging from 2 to 12 were evaluated in time by collecting UV–VIS spectra for compounds **5** and **7**. The existence of multiple species upon pH changes was evidenced by the presence of different absorption maxima for both compounds **5** and **7**, as shown in Figure 6.

On the basis of the UV–VIS spectra collected in time (presented in Supplementary Materials, Figures S25 and S26), several species from the network of chemical reactions responsible for the halochromic behavior were identified. The flavylium cation AH^+^ was found to be stable in time at pH values below 3 for compound **5** and below 2 for compound **7**. Their absorption maxima were found at 478 nm (compound **5**) and 456 nm (compound **7**). The quinoidal base A, with absorption bands at 530 nm for compound **5**, and 520 nm for compound **7**, was evidenced at pH values above 4 for compound **5** and above 3 for compound **7**. Both bands corresponding to the quinoidal bases of **5** and **7** presented two shoulders at 500 nm and 570 nm (compound **5**), and 500 nm and 560 nm (compound **7**). The quinoidal bases were relatively stable in the pH range 5 to 10 for up to 24 h, but with the increase in pH the stability decreases in time. At pH values between 5 and 9 (for compound **5**) and between 7 and 9 (for compound **7**), absorption bands situated at about 370 nm (**5**) and 360 nm (**7**), respectively, can correspond to the *cis*-chalcone Cc species. At higher pH values (8 to 9), *trans*-chalcone was formed with absorption bands situated at 400 nm (**5**) and 370 nm (**7**). At pH values above 11, the deprotonation of *trans*-chalcone occurred, which can be observed by the presence of a shoulder in the UV–VIS spectra at 460 nm (**5**) and 450 nm (**7**), respectively, which increased in time.

**Figure 7:**
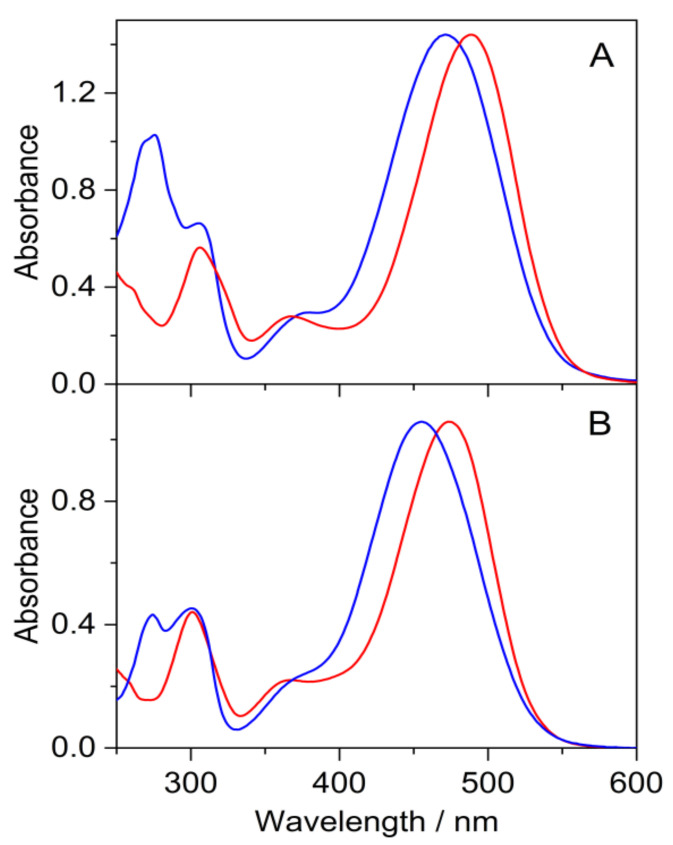
Absorption spectra of **5** (red line) and **7** (blue line) in methanol (**A**) and 0.01 M HCl aqueous solution (**B**).

The absorption spectra of **5** and **7** in methanol and 0.01 M HCl aqueous solutions (Figure 7) resembled that of flavylium compounds (AH**^+^**). The maximum of the long-wavelength band (λ_max_) of **5** showed 18 nm bathochromic shift in both solvents compared to the corresponding peak of **7** due to the stronger electron donating effect of the methoxy substituent in position 6. The change of the solvent from methanol to acidic water resulted in 16 nm blue-shift in λ_max_ for **5** and **7** alike.

At pH 2, the spectrum of **5** remained unchanged, but the band of **7** in the visible spectral range weakened, accompanied by the slight absorbance growth in the 310–390 nm domain within 22 h (Figure S27 in the Supplementary Materials). These alterations became more substantial at pH 3, where a fraction of **5** was also transformed within 22 h. The rise of the absorbance in the 310–390 nm range at the expense of the intensity of AH^+^ band indicated the formation of *cis*- and *trans*-chalcones (Cc and Ct).

**Figure 8:**
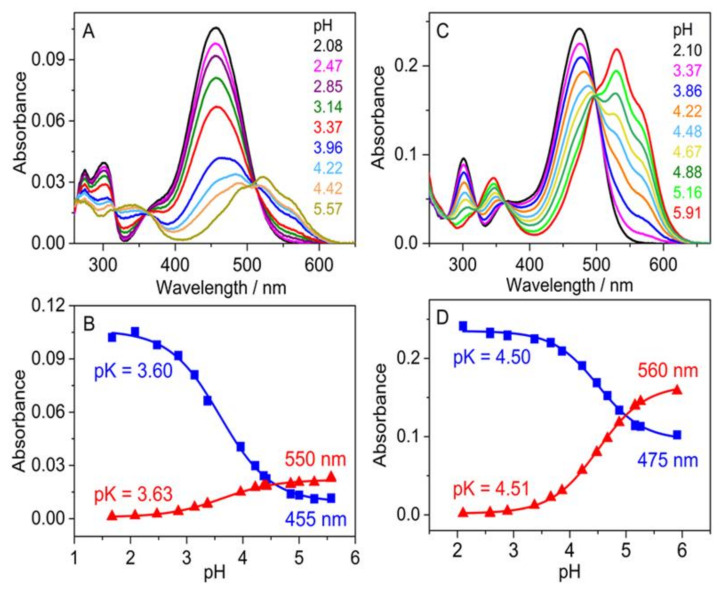
Effect of pH variation on the absorption spectrum of **7** (**A**) and **5** (**C**) in water. Absorbance of **7** (**B**) and **5** (**D**) solutions at two wavelengths as a function of pH. Measurements were performed immediately after pH adjustment.

Figure 8A,C shows the spectrum variations immediately after the addition of various amounts of NaOH to the strongly acidic aqueous solutions of flavylium compounds. The gradual vanishment of the original absorption of flavylium cations was coupled with the emergence of a structured new band in the 420–620 nm region due to the fast deprotonation-induced formation of the quinoidal base. Figure 8B,D presents the pH dependence of the absorbances (*A*) at two wavelengths. The experimental data were analyzed by the following relationship:

(1) $$A=\frac{A_{0}-A_{\infty }}{1+\exp[(pH-pK)\ln10}+A_{\infty }$$

where *pK* represents the negative logarithm of the equilibrium constant of the proton dissociation from AH^+^, whereas *A*_0_ and *A_∞_* are the absorbances at low and high pH values, respectively. The *pK* values obtained by the nonlinear least-squares fit of Equation (1) to the results measured at two wavelengths agreed well. Significantly lower acidity was found for **5** (*pK* = 4.51) than for **7** (*pK* = 3.61) due to the stronger electron donating effect of the methoxy substituent in positions 6.

**Figure 9:**
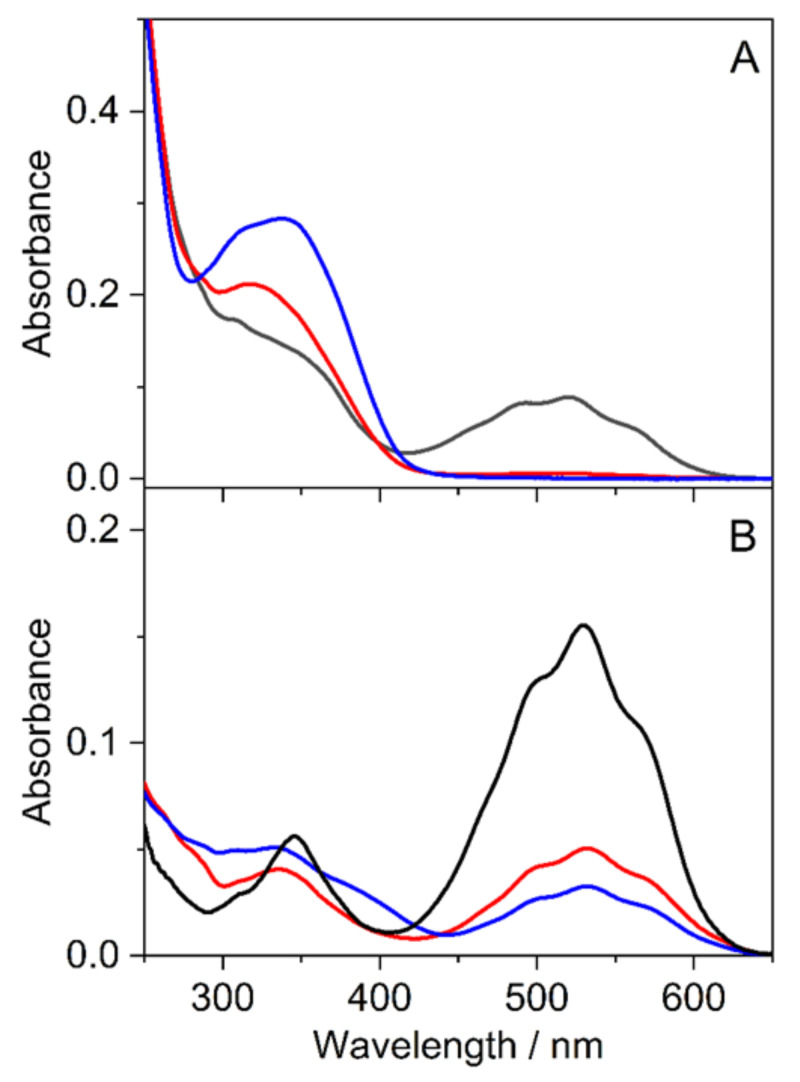
Absorption spectra of **7** (**A**) and **5** (**B**) immediately after the preparation of the samples in water (black lines) and in 20.84 mg/mL SBECD aqueous solution (red lines). Blue lines represent the spectra of the latter solutions in 3 h.

The effect of multianionic sulfobutylether-β-cyclodextrin (SBECD) and uncharged randomly methylated β-cyclodextrin (RAMEB) was studied in water, which had pH 5.6 because the dissolution of CO_2_ from air produces a small amount H_2_CO_3_ weak acid. Under this condition, both **5** and **7** were transformed to their deprotonated A form, showing the characteristic absorption in the 420–620 nm wavelength range. In the presence of 20.84 mg/mL SBECD, this band of **7** almost completely vanished, and an absorption peaking at 317 nm appeared (Figure 9A).

The changes imply that the preferential binding of Cc to SBECD shifts the three-step equilibria between A and Cc (Figure 9) toward the production of the latter species. The intermediate B may also be confined in SBECD to a smaller extent. A previous study of a structurally similar anthocyanin, 7-β-D-glucopyranosyloxy-4′-hydroxyflavylium complexation with SBECD, demonstrated the largest affinity of the Cc form to the host macrocycle [41]. We found considerable alteration of the spectrum when the solution containing 20.84 mg/mL SBECD was kept for 3 h in the dark. The changes are attributed to the slow isomerization of Cc into the thermodynamically more stable Ct, which is probably also capable of the encapsulation in SBECD. Similar behavior was observed for 5, but significant A absorbance remained in the presence of SBECD even after staying in the dark for 3 h (Figure 9B). The spectral alterations barely differed when 11.43 mg/mL RAMEB served as a host instead of SBECD (Figure S28 in Supplementary Materials), suggesting that the charge of the macrocycle plays a minor role in the complexation-induced transformations.

### 3.4. Cytotoxicity of Flavylium Complexes with Cyclodextrins

**Figure 10:**
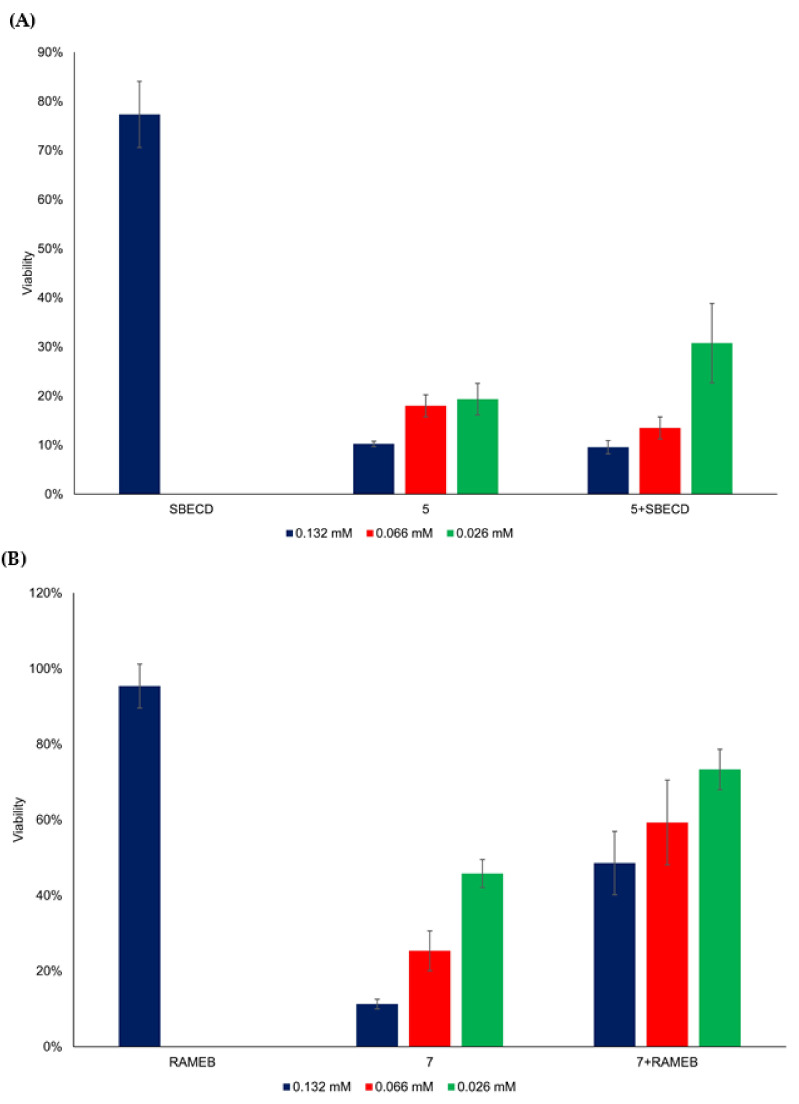
Viability (%) of HepG2 cells as a function of flavylium compounds **5** (**A**) and **7** (**B**), and their cyclodextrin complex concentration with SBECD and RAMEB, respectively.

The main technological advantage of the macrocyclic oligosaccharides known as cyclodextrins, which are accessible in great structural variety, is the improvement of the solubility and bioavailability of bioactive molecules with low solubility in water [42,43]. On the basis of the formation of inclusion complexes and shifting the thermodynamic equilibria of the studied synthetic anthocyanidins toward the chalcones, as demonstrated in the previous section, the in vitro cytotoxicity of the selected flavylium complexes with the cyclodextrin derivatives SBECD and RAMEB was also investigated in HepG2 cells (Figure 10). SBCED was selected for molecular encapsulation of **5** and RAMEB for **7** because of the water solubility increase upon complexation, as was shown in the Materials and Methods section. The cyclodextrin concentration (*m*/*v*) was 10-fold higher than the flavylium concentration. The complexation of **7** with RAMEB reduced its cytotoxic effect substantially; however, SBECD did not change the cytotoxicity of **5** significantly (Figure 10).

In the complexed samples (5 + SBECD, 7 + RAMEB), a significant difference (*p* < 0.01) was found between 132 μM and 26 μM (5 + SBECD) and between 66 μM and 26 μM (7 + RAMEB) concentration levels (Table S3). A significant difference (*p* < 0.01) was detected in the case of comparing the flavylium compounds with complexed ones, grouped by concentration levels (Table S4). Consequently, compound **5** species exerted the most efficient anticancer effect among the eight studied flavylium compounds, and it also kept its influence after complexation with a water-soluble cyclodextrin (SBECD). The pH of blood plasma is slightly basic, around 7.4. As already mentioned, at neutral and slightly basic pH values, the basic species, quinoidal base, hemiketal, and *cis*- and *trans*-chalcones, are predominant among the multistate of different molecules present in the system. Their relative fractions are also dependent on the substitution pattern of the flavylium cation [44]. Concerning the investigated synthetic anthocyanidins, the absorption spectra (Figure 9) demonstrated that in the case of the inclusion complex of soluble cyclodextrin SBECD with the compound showing the most important anticancer effect **5**, the quinoidal base was present in significant amount, together with the *cis*- and *trans*-chalcones. Obviously, both the formation of the inclusion complex and the cytostatic effect are the result of a complex equilibrium between all basic species, also influenced by the position of the methoxy substituent in the flavylium cation. On the basis of the excellent study of Gago et al. [34], we can presume that also in the case of our compounds, the formation of the complex with the water-soluble cyclodextrin derivative SBCED led to shifting of the equilibrium towards the *trans*-chalcone form, and the anticancer activity is mainly due to this complex. Further studies will better elucidate this influence.

## 4. Conclusions

The chemical synthesis of new anthocyanidins with hydroxy or methoxy group(s) in the aromatic ring was accomplished from 4′-hydroxy-3′,5′-dimethoxyacetophenone and 2-hydroxybenzaldehyde derivatives. The assessment of anticancer activities on HepG2 (hepatocellular carcinoma cells) and HCT116 (colon cells) showed the highest efficiency for the flavylium derivatives, holding methoxy groups in the flavylium cation (compounds **5** and **7**), even at low concentrations (26 μM). Formation of inclusion complex of compound **5** with the cyclodextrin derivative SBECD led to 1.5-fold increase in the water solubility, preserving 70% of the cytotoxic effect on HepG2 cells. Other post-encapsulation viability studies on various cell lines, as well as anti-tumor experiments in vivo, will be carried out in order to sustain and develop these findings.

## Data Availability

The data presented in this study are available on request from the corresponding authors.

## Supplementary Materials

The following supporting information can be downloaded at www.mdpi.com/article/10.3390/ijms23158103/s1.
