# Supplementary material for: Halochromic Behavior and Anticancer Effect of New Synthetic Anthocyanidins Complexed with β-Cyclodextrin Derivatives
Source: Int J Mol Sci. 2022 Jul 22;23(15):8103. doi: 10.3390/ijms23158103 (PMC9330608; doi:10.3390/ijms23158103)
Supplement: Supplementary file 1 [file ijms-23-08103-s001.zip › ijms-1784726-supplementary.pdf]

Halochromic behavior and anticancer effect of new synthetic anthocyanidins complexed with β-cyclodextrin derivatives

Iulia Păușescu [1], Izolda Kántor [2,3], György Babos [3], Zoltán May [2], Andrea Fodor-Kardos [2,3], Zsombor Miskolczy [2], László Biczók [2], Francisc Péter [1,4], Mihai Medeleanu [1*], Tivadar Feczkó [2,3*]

[1] University Politehnica Timişoara, Faculty of Industrial Chemistry and Environmental Engineering, C. Telbisz 6, 300001 Timişoara, Romania;

[2] Institute of Materials and Environmental Chemistry, Research Centre for Natural Sciences, Magyar tudósok körútja 2, H-1117, Budapest, Hungary;

[3] Faculty of Engineering, University of Pannonia, Egyetem u. 10, H-8200, Veszprém, Hungary;

[4] University Politehnica Timişoara, Research Institute for Renewable Energies, G. Muzicescu 138, 300501, Timişoara, Romania;

Supplementary material

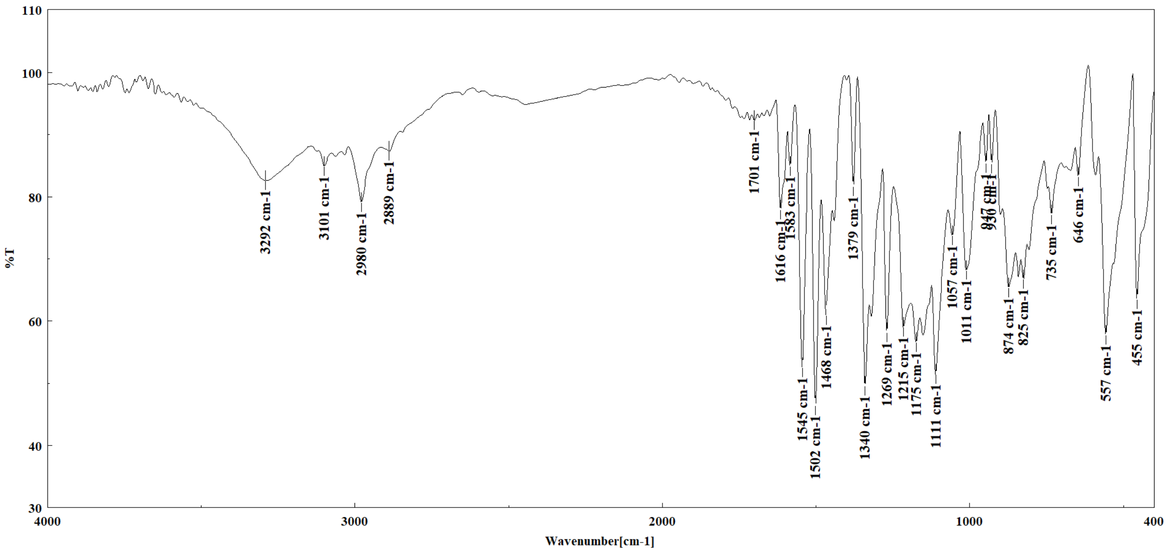

Figure S1. FT-IR spectrum of compound **5**

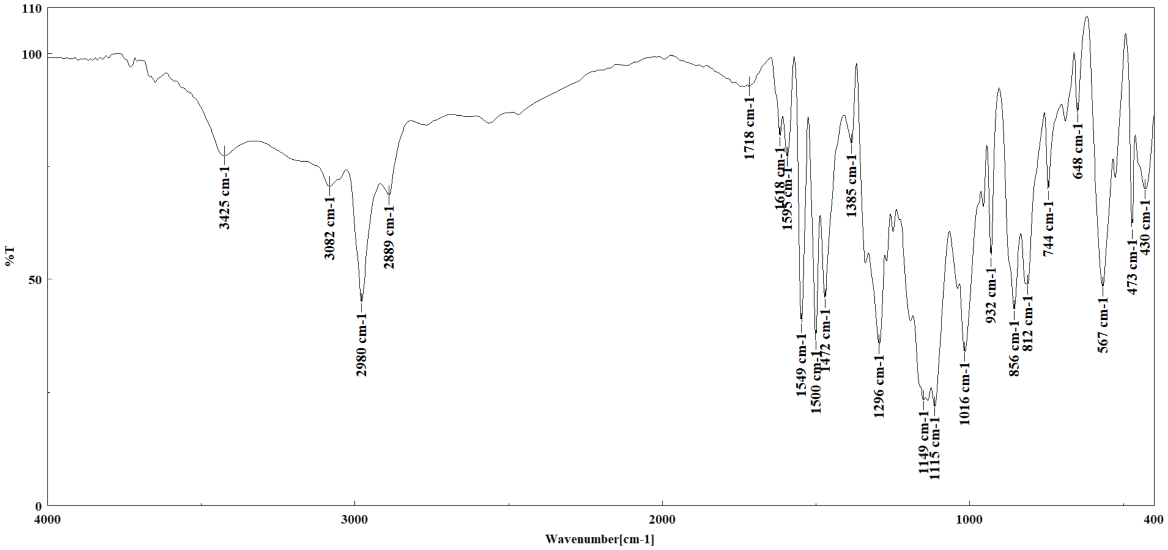

Figure S2. FT-IR spectrum of compound **1**

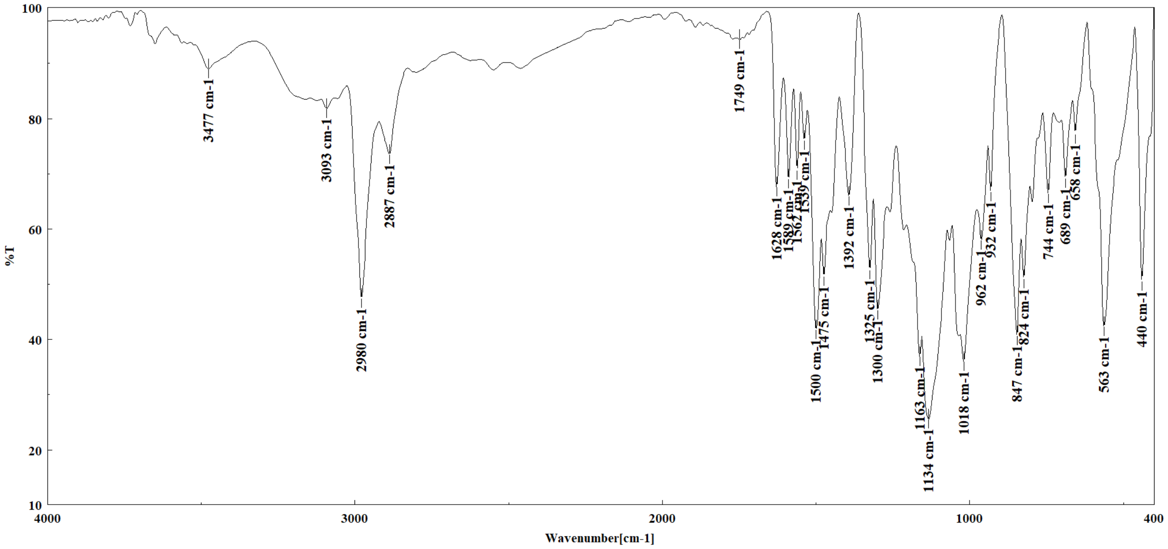

Figure S3. FT-IR spectrum of compound **2**

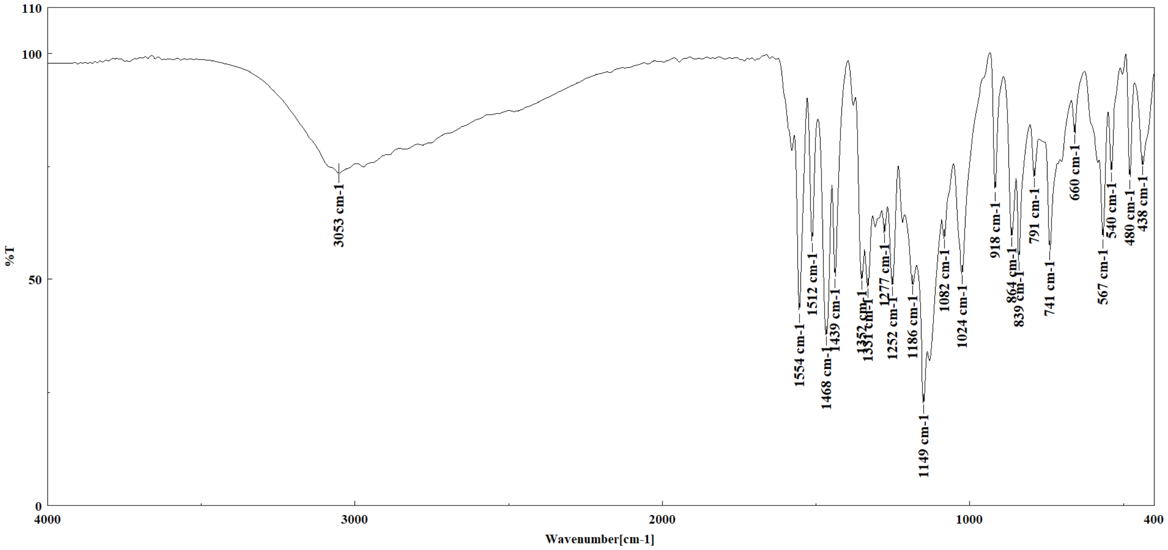

Figure S4. FT-IR spectrum of compound **3**

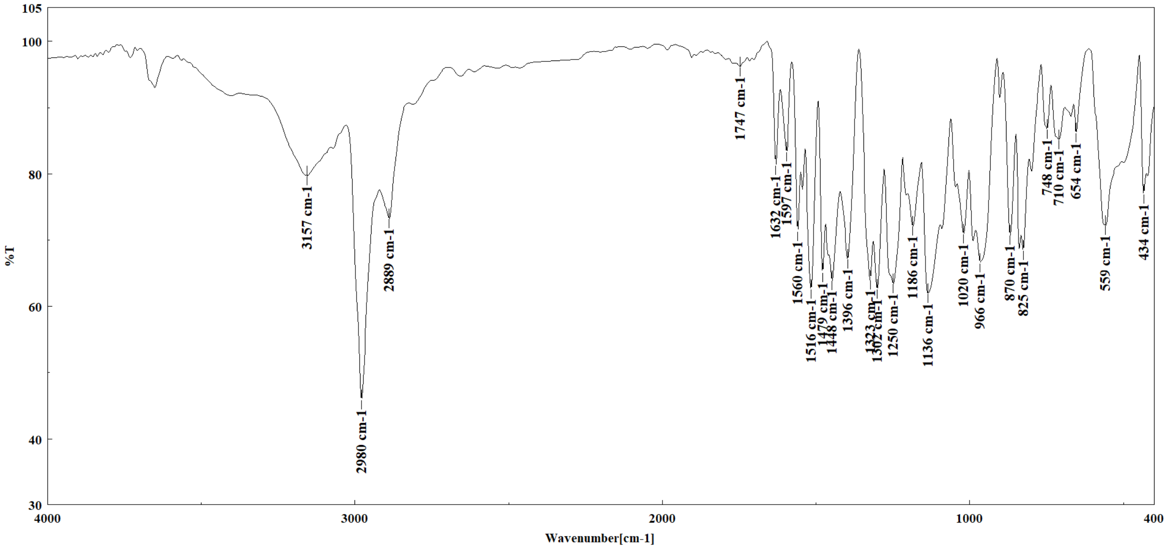

Figure S5. FT-IR spectrum of compound **4**

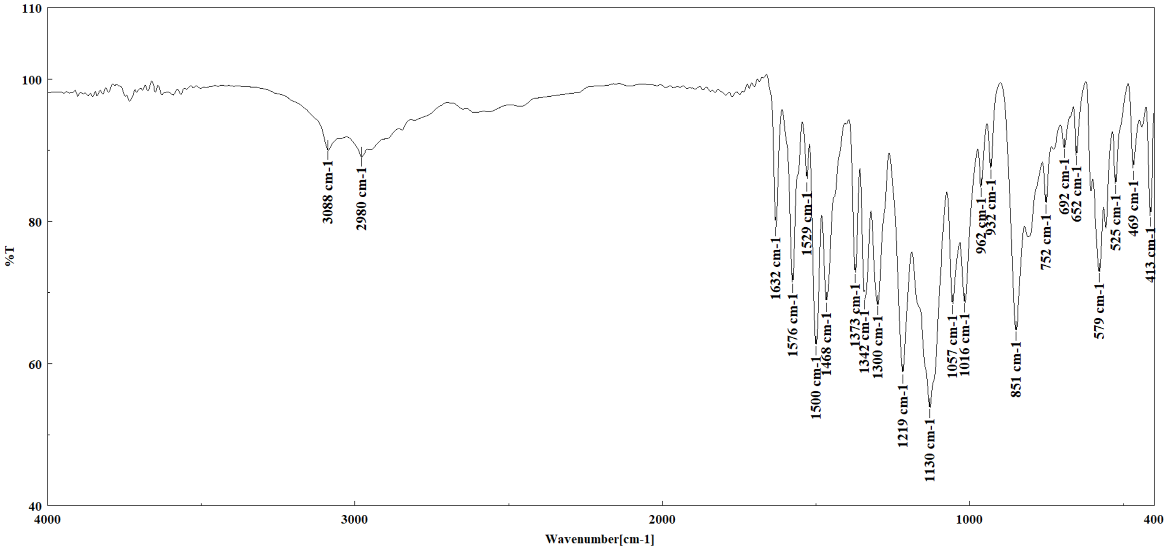

Figure S6. FT-IR spectrum of compound **6**

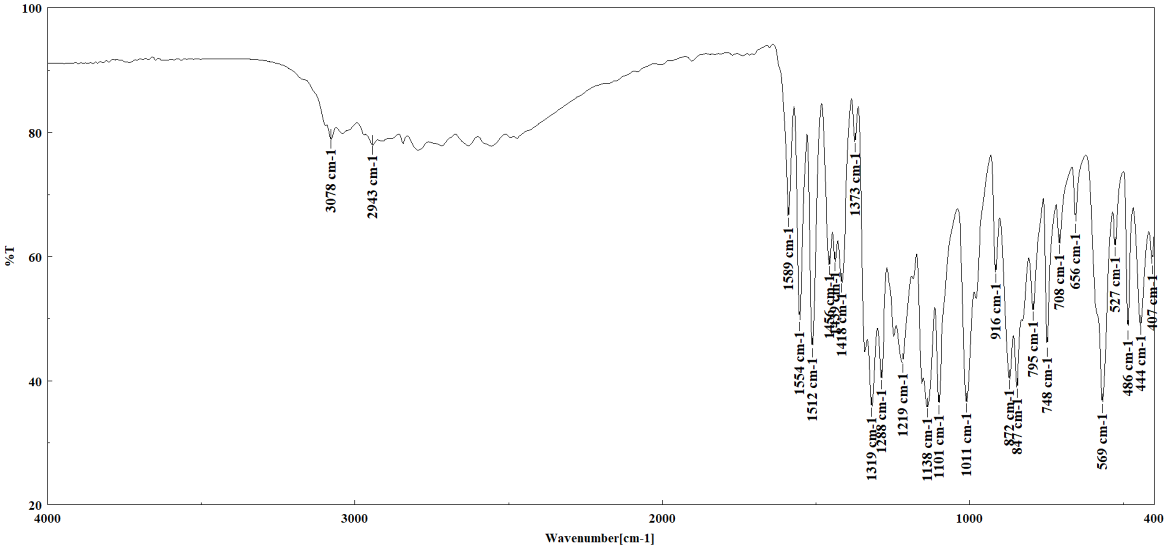

Figure S7. FT-IR spectrum of compound **7**

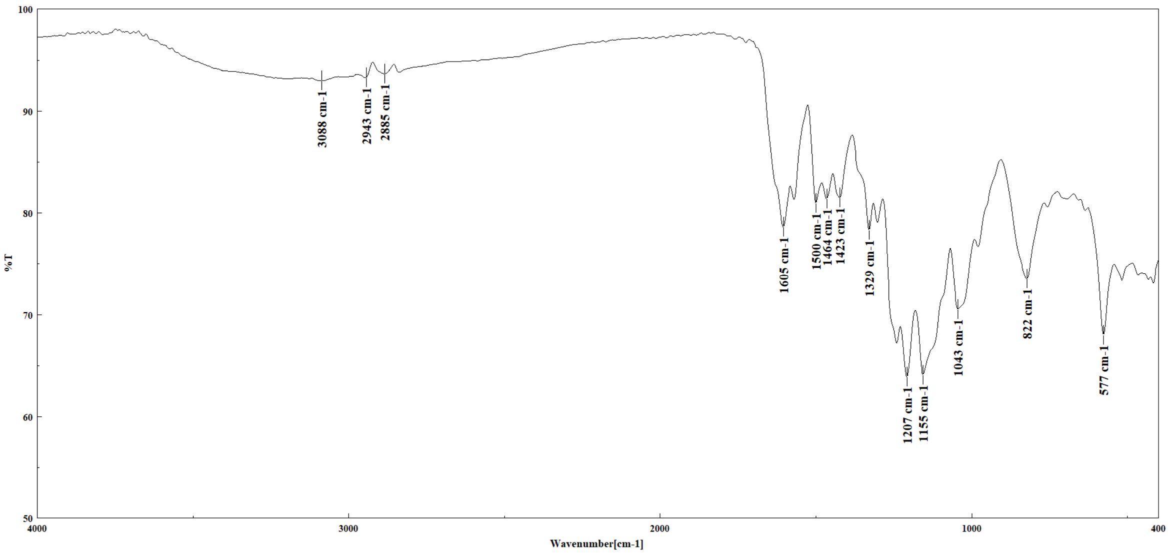

Figure S8. FT-IR spectrum of compound **8**

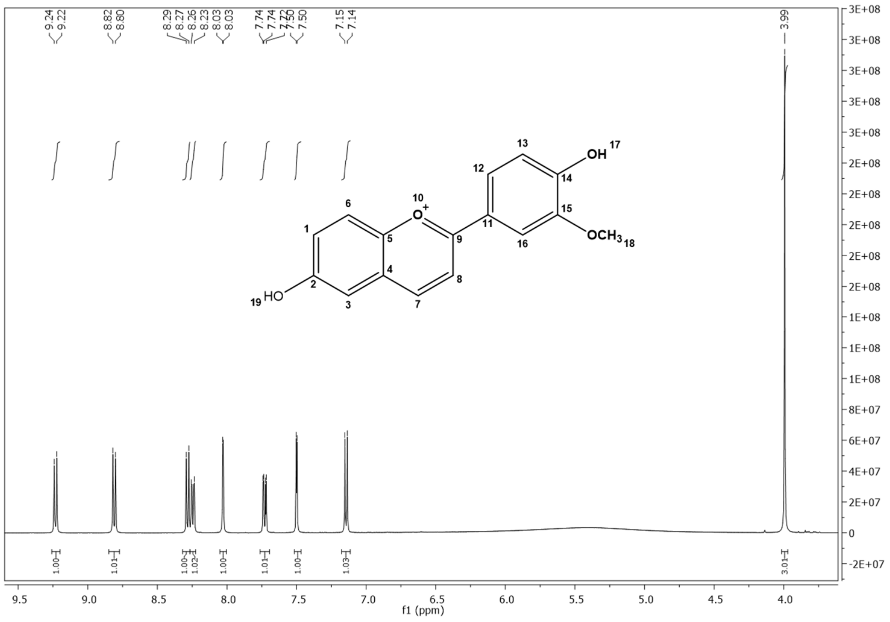

Figure S9. ^{1}H-NMR spectrum of compound **1**

^{1}**H-NMR** (500 MHz, DMSO-d_6, δ ppm): 9.23 (d, J = 9.2 Hz, 1H, **H7**), 8.81 (d, J = 9.2 Hz, 1H, **H8**), 8.28 (d, J = 9.3 Hz, 1H, **H6**), 8.24 (d, J = 10.8 Hz, 1H, **H12**), 8.03 (d, J = 2.1 Hz, 1H, **H13**), 7.73 (dd, J = 9.2, 2.9 Hz, 1H, **H1**), 7.50 (d, J = 2.9 Hz, 1H, **H3**), 7.14 (d, J = 8.6 Hz, 1H, **H16**), 3.99 (s, 3H, **H18**).

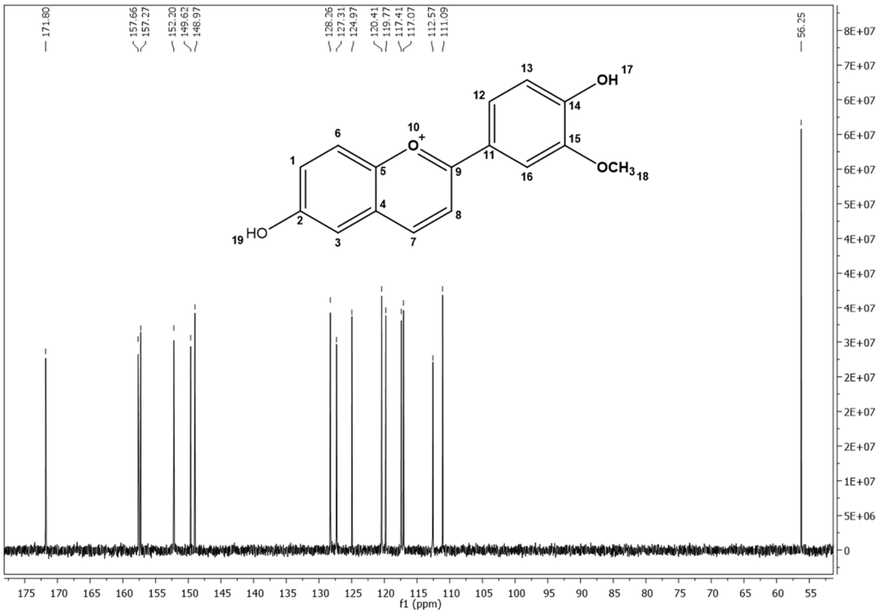

Figure S10. ^{13}C-NMR spectrum of compound **1**

^{13}C-NMR (125 MHz, DMSO-*d$_6$*, δ ppm): 171.8 (**C9**); 157.6 (**C15**); 157.2 (**C2**); 152.2(**C7**); 149.6 (**C14**); 148.9 (**C5**); 128.2 (**C1**); 127.3 (**C12**); 124.9 (**C4**); 120.4 (**C6**); 119.7 (**C11**); 117.4 (**C8**); 117.0 (**C16**); 112.5 (**C13**); 111.0 (**C3**); 56.2 (**C18**).

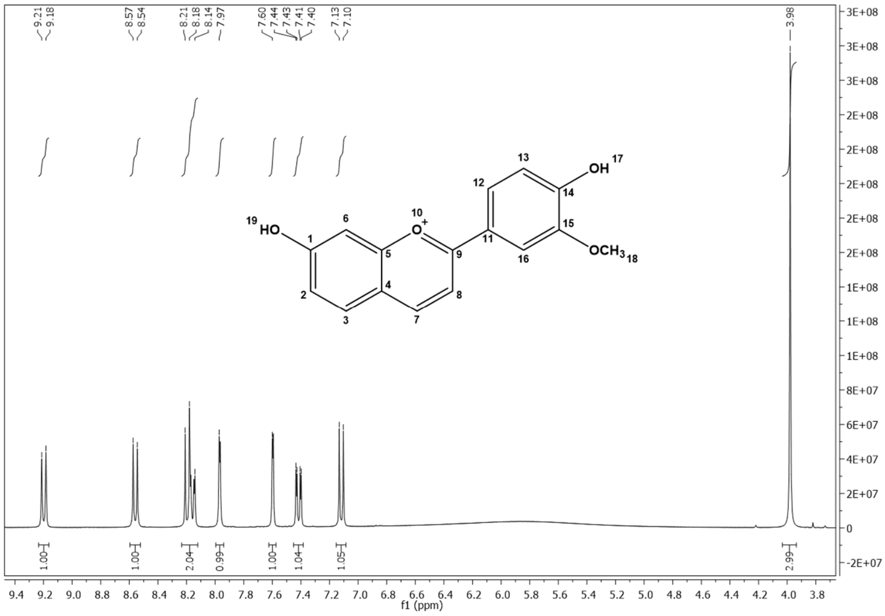

Figure S11. ^{1}H-NMR spectrum of compound **2**

^{1}H-NMR (300 MHz, DMSO-*d$_6$*, δ ppm): 9.20 (d, *J* = 8.8 Hz, 1H, **H7**), 8.56 (d, *J* = 8.8 Hz, 1H, **H8**), 8.18 (t, *J* = 10.3 Hz, 2H, **H3**, **H12**), 7.97 (s, 1H, **H16**), 7.60 (s, 1H, **H6**), 7.42 (dd, *J* = 8.9, 2.2 Hz, 1H, **H13**), 7.12 (d, *J* = 8.6 Hz, 1H, **H2**), 3.98 (s, 3H, **H18**).

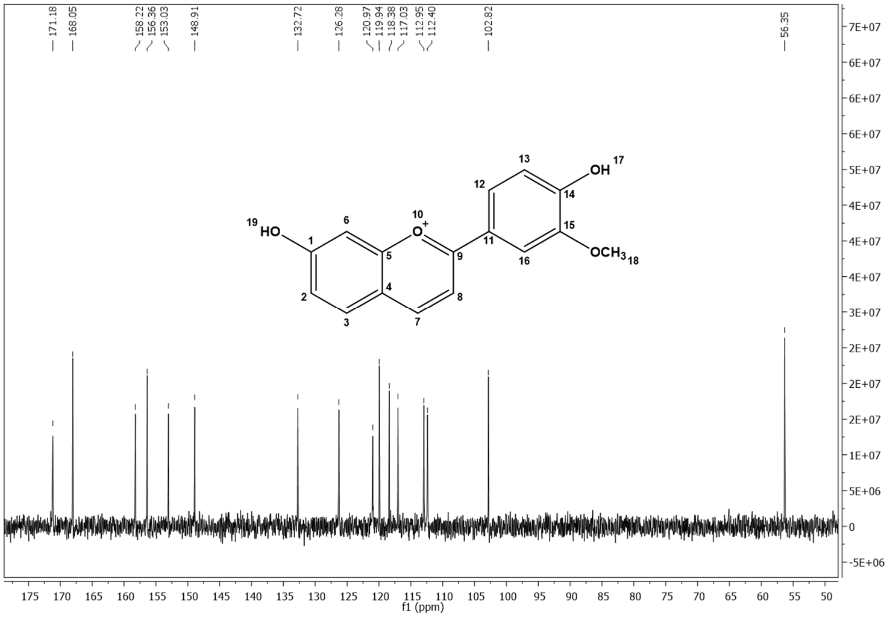

Figure S12. ^{13}C-NMR spectrum of compound **2**

^{13}C-NMR (75 MHz, DMSO-d_6, δ ppm): 171.1 (**C9**); 168.0 (**C1**); 158.2 (**C5**); 156.3 (**C14**); 153.0 (**C7**); 148.9 (**C15**); 132.7 (**C3**); 126.2 (**C12**); 120.9 (**C13**); 119.3 (**C4**); 118.3 (**C11**); 117.0 (**C2**); 112.9 (**C8**); 112.4 (**C16**); 102.8 (**C6**); 56.3 (**C18**).

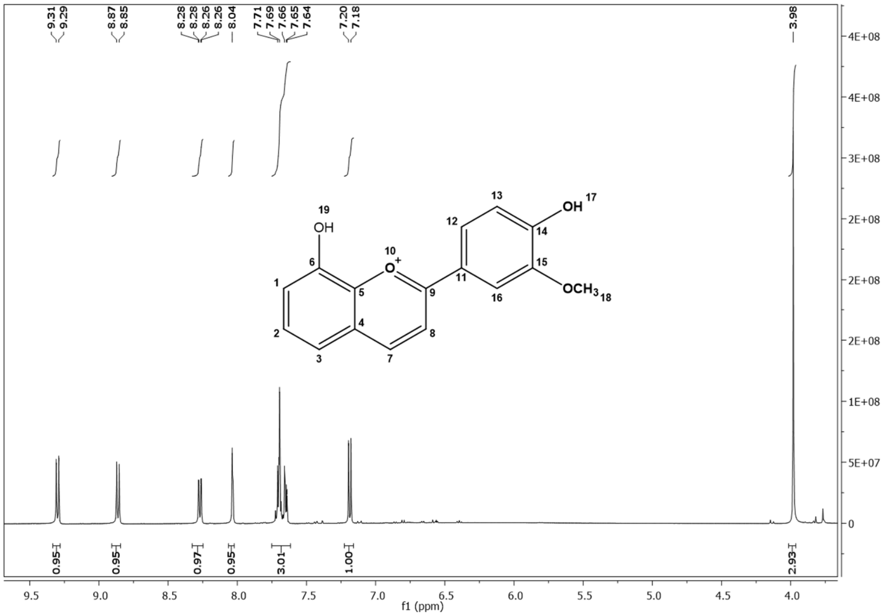

Figure S13. ^{1}H-NMR spectrum of compound **3**

^{1}H-NMR (500 MHz, DMSO-d_6, δ ppm): 9.30 (d, J = 9.2 Hz, 1H, **H7**), 8.86 (d, J = 9.2 Hz, 1H, **H8**), 8.27 (dd, J = 8.7, 2.2 Hz, 1H, **H12**), 8.04 (s, 1H, **H16**), 7.75 – 7.61 (m, 3H, **H1**, **H2**, **H3**), 7.19 (d, J = 8.7 Hz, 1H, **H13**), 3.98 (s, 3H, **H18**).

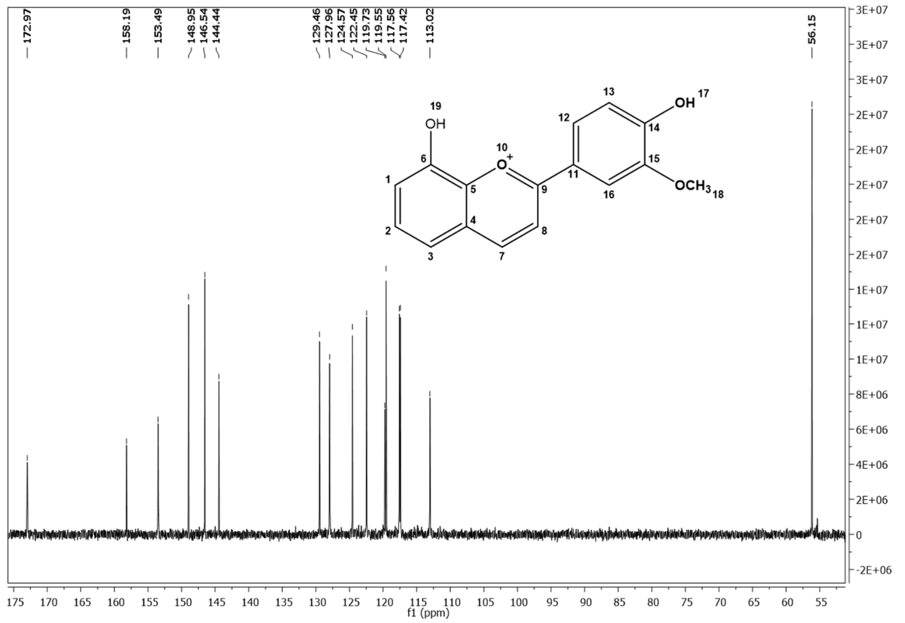

Figure S14. ^{13}C-NMR spectrum of compound **3**

^{13}C-NMR (125 MHz, DMSO-*d₆*, δ ppm): 172.9 (**C9**); 158.1 (**C14**); 153.4 (**C7**); 148.9 (**C15**); 146.5 (**C5**); 144.4 (**C6**); 129.4 (**C2**); 127.9 (**C12**); 124.5 (**C4**); 122.4 (**C3**); 119.7 (**C11**); 119.5 (**C1**); 117.5 (**C8**); 117.4 (**C13**); 113.0 (**C16**); 56.1 (**C18**).

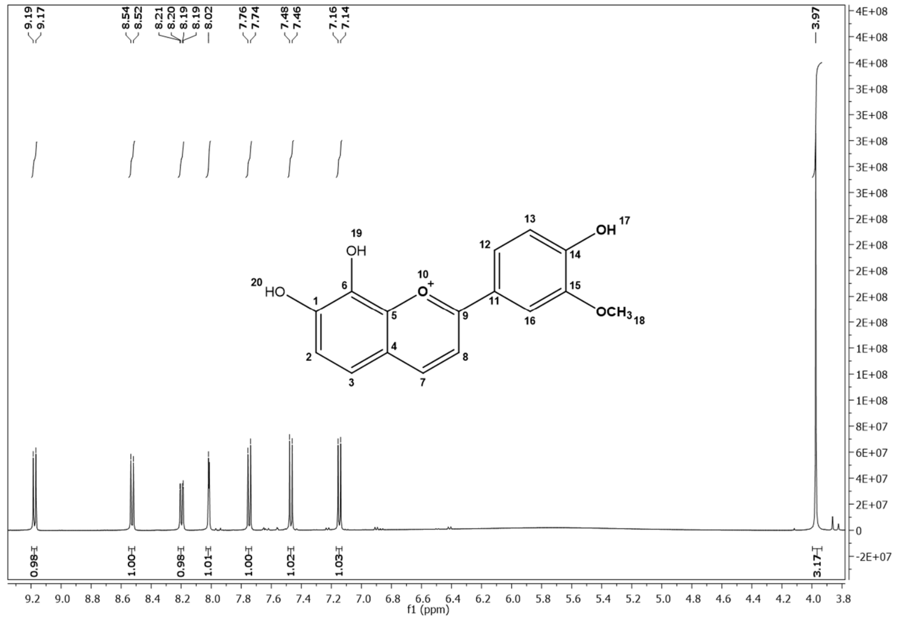

Figure S15. ^{1}H-NMR spectrum of compound **4**

^{1}H-NMR (500 MHz, DMSO-*d₆*, δ ppm): 9.18 (d, *J* = 8.9 Hz, 1H, **H7**), 8.53 (d, *J* = 8.8 Hz, 1H, **H8**), 8.20 (dd, *J* = 8.6, 2.2 Hz, 1H, **H12**), 8.02 (s, 1H, **H16**), 7.75 (d, *J* = 8.8 Hz, 1H, **H3**), 7.47 (d, *J* = 8.8 Hz, 1H, **H2**), 7.15 (d, *J* = 8.6 Hz, 1H, **H13**), 3.97 (s, 3H, **H18**).

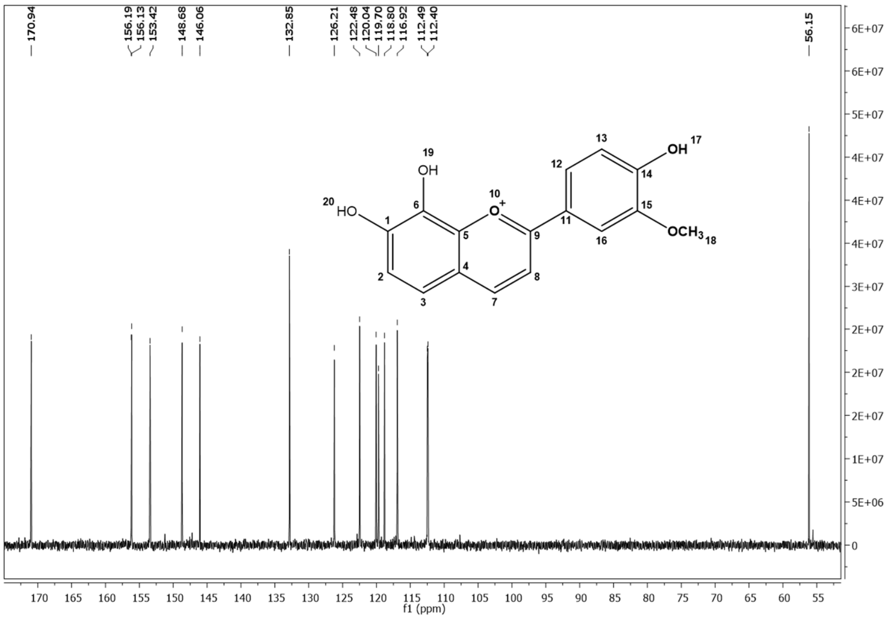

Figure S16. ^{13}C-NMR spectrum of compound **4**

^{13}**C-NMR** (125 MHz, DMSO-d_6, δ ppm): 170.9 (**C9**); 156.2 (**C15**); 156.1 (**C1**); 156.1 (**C14**); 153.4 (**C7**); 148.6 (**C14**); 146.0 (**C5**); 132.8 (**C6**); 126.2 (**C12**); 122.4 (**C3**); 120.0 (**C4**); 119.7 (**C2**); 118.8 (**C11**); 116.9 (**C13**); 112.5 (**C16**); 112.4 (**C8**); 56.1 (**C18**).

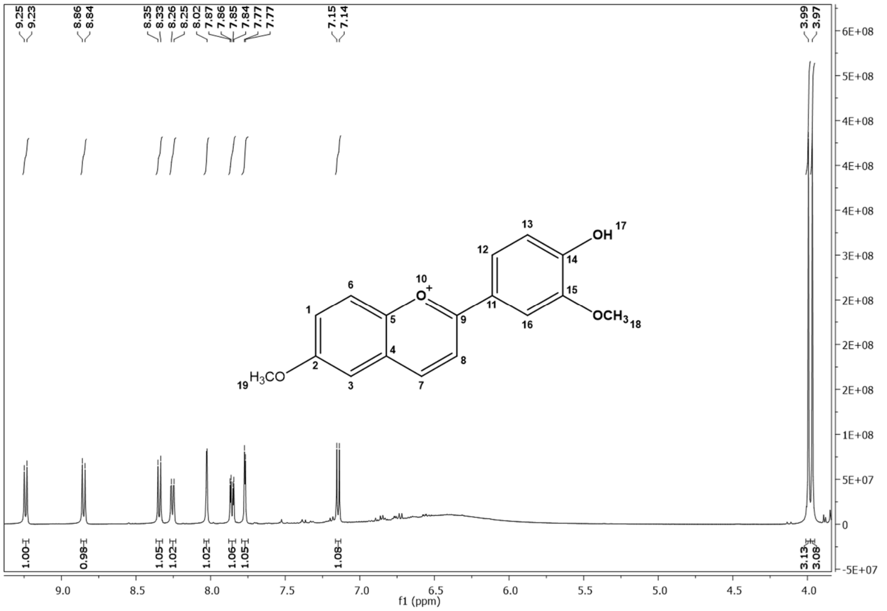

Figure S17. ^{1}H-NMR spectrum of compound **5**

^{1}**H-NMR** (500 MHz, DMSO-d_6, δ ppm): 9.24 (d, *J* = 9.2 Hz, 1H, **H7**), 8.85 (d, *J* = 9.2 Hz, 1H, **H8**), 8.34 (d, *J* = 9.3 Hz, 1H, **H6**), 8.25 (d, *J* = 8.6 Hz, 1H, **H12**), 8.02 (s, 1H, **H16**), 7.86 (dd, *J* = 9.3, 3.0 Hz, 1H, **H1**), 7.77 (d, *J* = 2.9 Hz, 1H, **H3**), 7.15 (d, *J* = 8.6 Hz, 1H, **H13**), 3.99 (s, 3H, **H18**), 3.97 (s, 3H, **H19**).

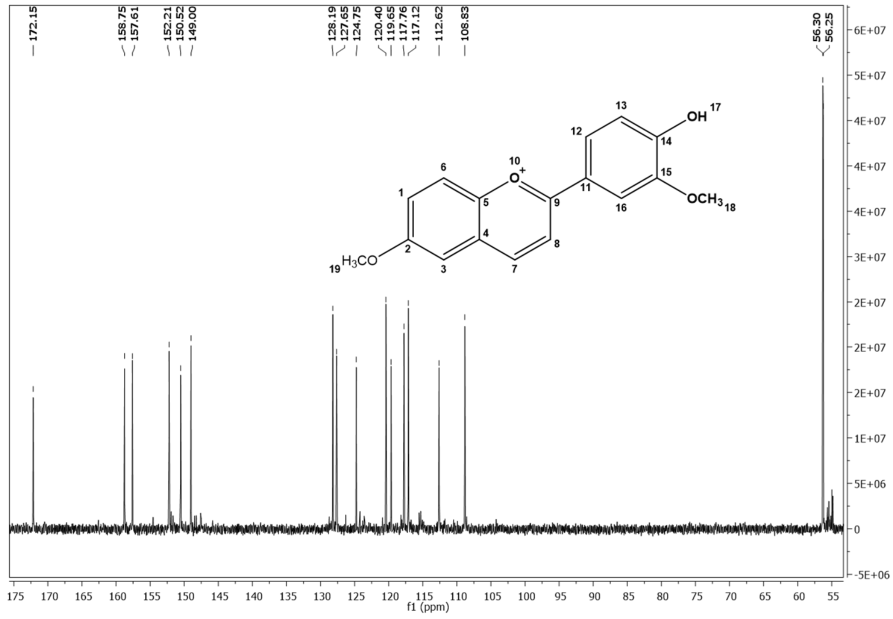

Figure S18. ^{13}C-NMR spectrum of compound **5**

^{13}C-NMR (125 MHz, DMSO-d_6, δ ppm): 172.1 (C**9**); 158.7 (C**2**); 157.6 (C**14**); 152.2 (C**7**); 150.5 (C**5**); 149.0 (C**15**); 128.1 (C**12**); 127.6 (C**1**); 124.7 (C**4**); 120.4 (C**6**); 119.6 (C**11**); 117.7 (C**8**); 117.1 (C**13**); 112.6 (C**16**); 108.8 (C**3**); 56.3 (C**18**); 56.2 (C1**9**).

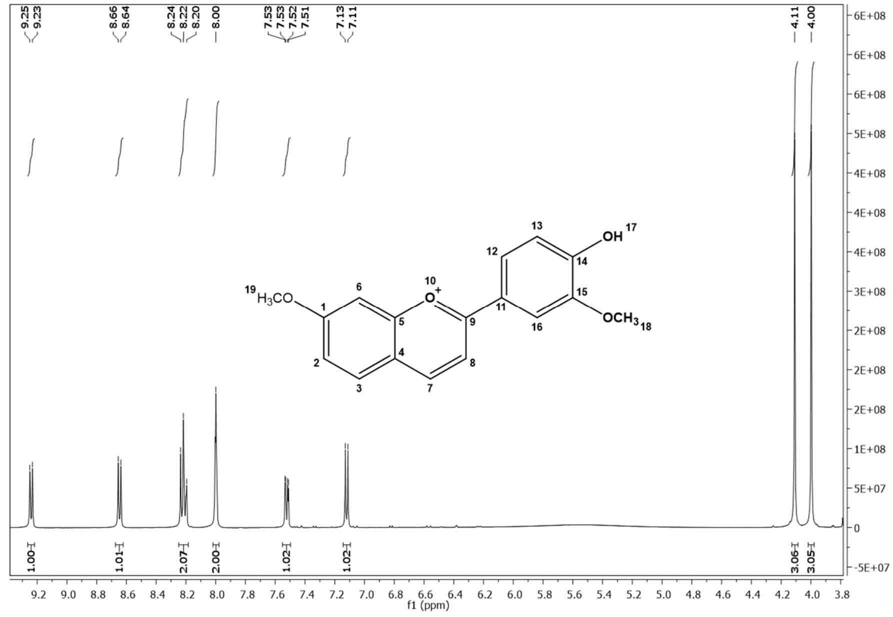

Figure S19. ^{1}H-NMR spectrum of compound **6**

^{1}H-NMR (500 MHz, DMSO-d_6, δ ppm): 9.24 (d, *J* = 8.9 Hz, 1H, **H7**), 8.65 (d, *J* = 8.9 Hz, 1H, **H8**), 8.22 (t, *J* = 10.1 Hz, 2H, **H3**, **H13**), 8.00 (s, 2H, **H6**, **H16**), 7.52 (dd, *J* = 9.0, 2.4 Hz, 1H, **H12**), 7.12 (d, *J* = 8.6 Hz, 1H, **H2**), 4.11 (s, 3H, **H18**), 4.00 (s, 3H, **H19**).

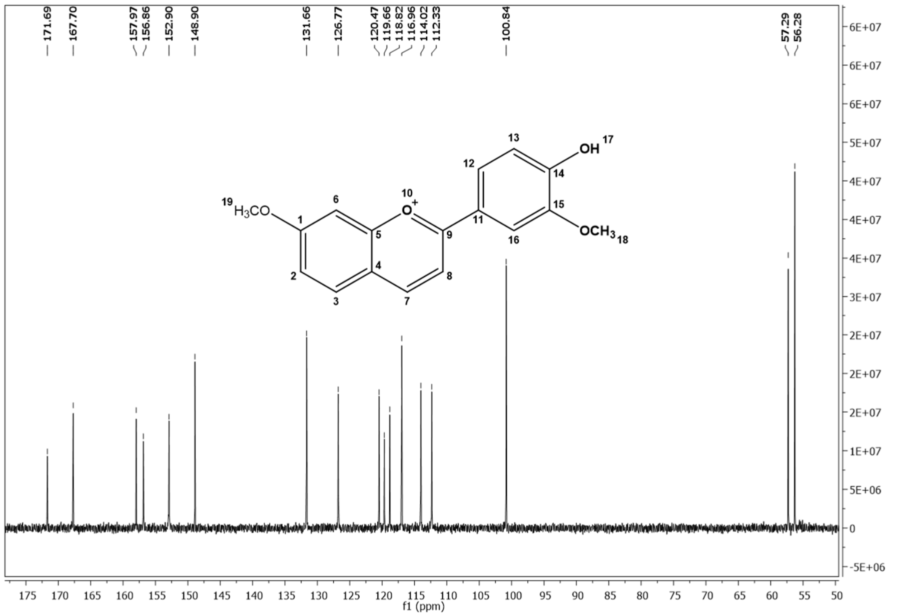

Figure S20. ^{13}C-NMR spectrum of compound **6**

^{13}C-NMR (125 MHz, DMSO-*d*$_6$, δ ppm): 171.6 (**C9**); 167.7 (**C15**); 157.9 (**C14**); 156.8 (**C5**); 152.9 (**C7**); 148.9 (**C1**); 131.6 (**C3**); 126.7 (**C13**); 120.4 (**C12**); 119.6 (**C11**); 118.8 (**C4**); 116.9 (**C2**); 114.0 (**C8**); 112.3 (**C16**); 100.8 (**C6**); 57.2 (**C18**); 56.2 (**C19**).

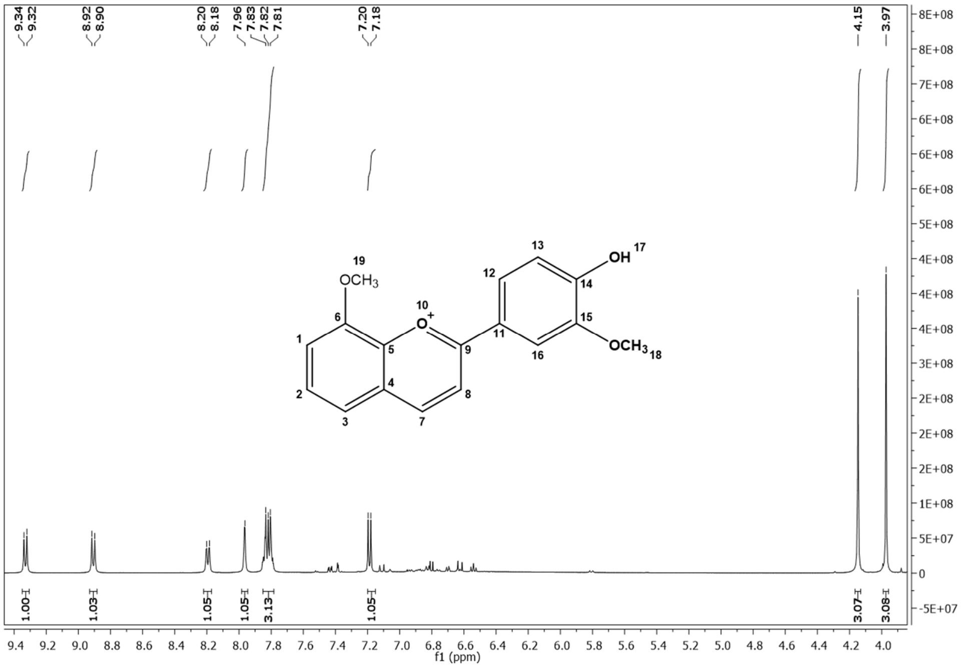

Figure S21. ^{1}H-NMR spectrum of compound **7**

^{1}H-NMR (500 MHz, DMSO-*d*$_6$, δ ppm): 9.33 (d, *J* = 9.2 Hz, 1H, H7), 8.91 (d, *J* = 9.2 Hz, 1H, **H8**), 8.19 (d, *J* = 8.6 Hz, 1H, **H12**), 7.96 (s, 1H, **H16**), 7.85 – 7.78 (m, 3H, **H1**, **H2**, **H3**), 7.19 (d, *J* = 8.6 Hz, 1H, **H13**), 4.15 (s, 3H, **H18**), 3.97 (s, 3H, **H19**).

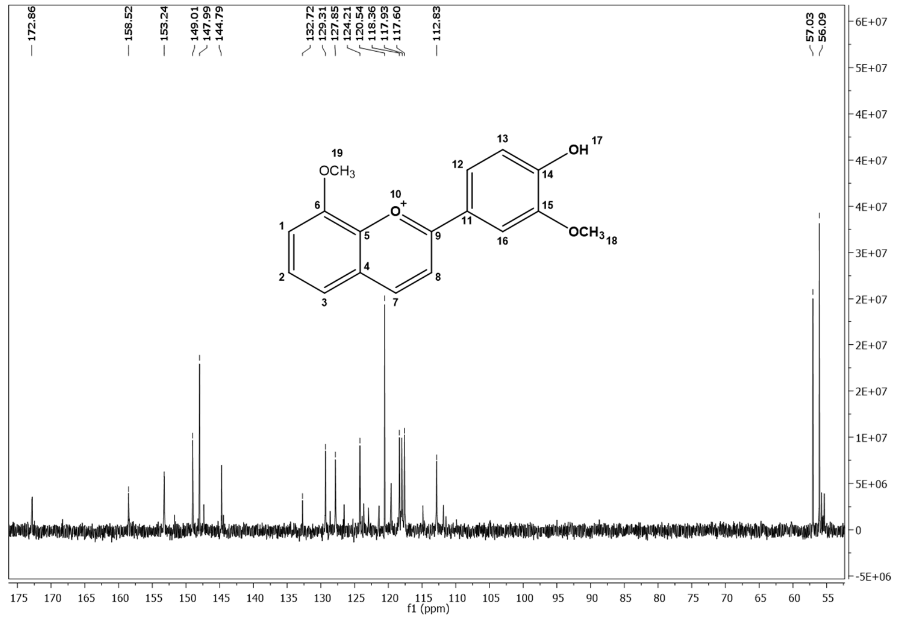

Figure S22. ^{13}C-NMR spectrum of compound **7**

^{13}C-NMR (125 MHz, DMSO-*d$_6$*, δ ppm): 172.8 (**C9**); 158.5 (**C14**); 153.2 (**C7**); 149.0 (**C15**); 147.7 (**C5**); 144.7 (**C6**); 132.7 (**C4**); 129.3 (**C2**); 127.8 (**C12**); 124.2 (**C11**); 120.5 (**C3**); 118.3 (**C1**); 117.9 (**C8**); 117.6 (**C13**); 112.8 (**C16**); 57.0 (**C18**); 56.0 (**C19**).

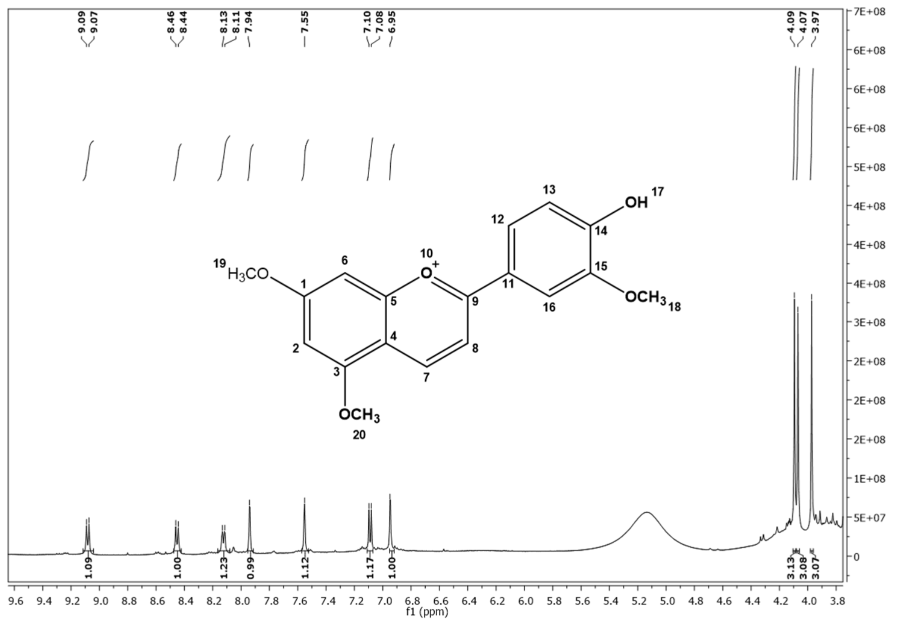

Figure S23. ^{1}H-NMR spectrum of compound **8**

^{1}H-NMR (500 MHz, DMSO-*d$_6$*, δ ppm): 9.08 (d, *J* = 8.9 Hz, 1H, **H7**), 8.45 (d, *J* = 8.9 Hz, 1H, **H8**), 8.12 (d, *J* = 8.5 Hz, 1H, **H12**), 7.94 (s, 1H, **H16**), 7.55 (s, 1H, **H6**), 7.09 (d, *J* = 8.6 Hz, 1H, **H13**), 6.95 (s, 1H, **H2**), 4.09 (s, 3H, **H19**), 4.07 (s, 3H, **H20**), 3.97 (s, 3H, **H18**).

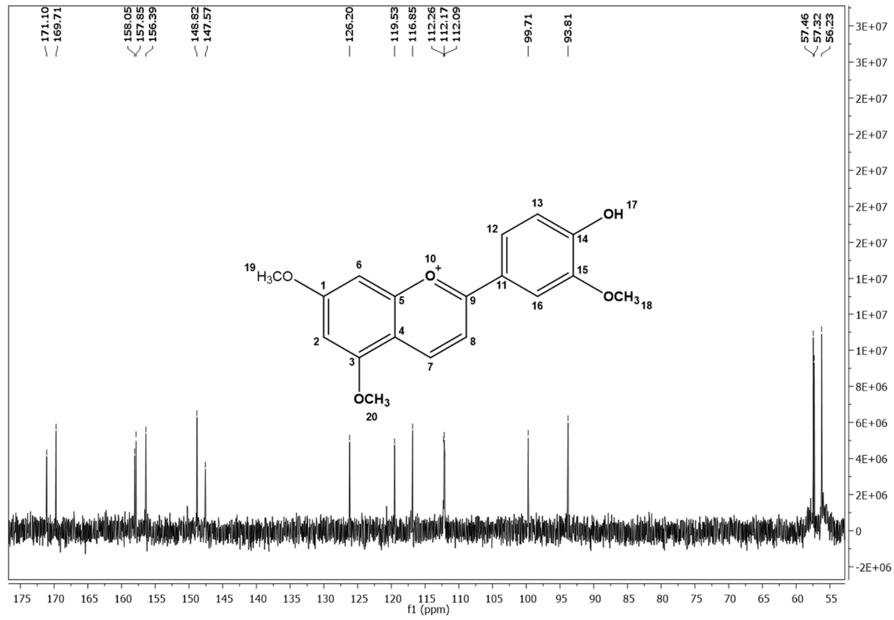

Figure S24. ^{13}C-NMR spectrum of compound **8**

^{13}C-NMR (125 MHz, DMSO-*d*$_6$, δ ppm): 171.1 (**C9**); 169.7 (**C19**); 158.0 (**C3**); 157.8 (**C14**); 156.3 (**C5**); 148.8 (**C15**); 147.5 (**C7**); 126.2 (**C12**); 119.5 (**C11**); 116.8 (**C13**); 112.2 (**C8**); 112.1 (**C16**); 112.0 (**C4**); 99.7 (**C2**); 93.8 (**C6**); 57.4 (**C19**); 57.3 (**C20**); 56.2 (**C18**).

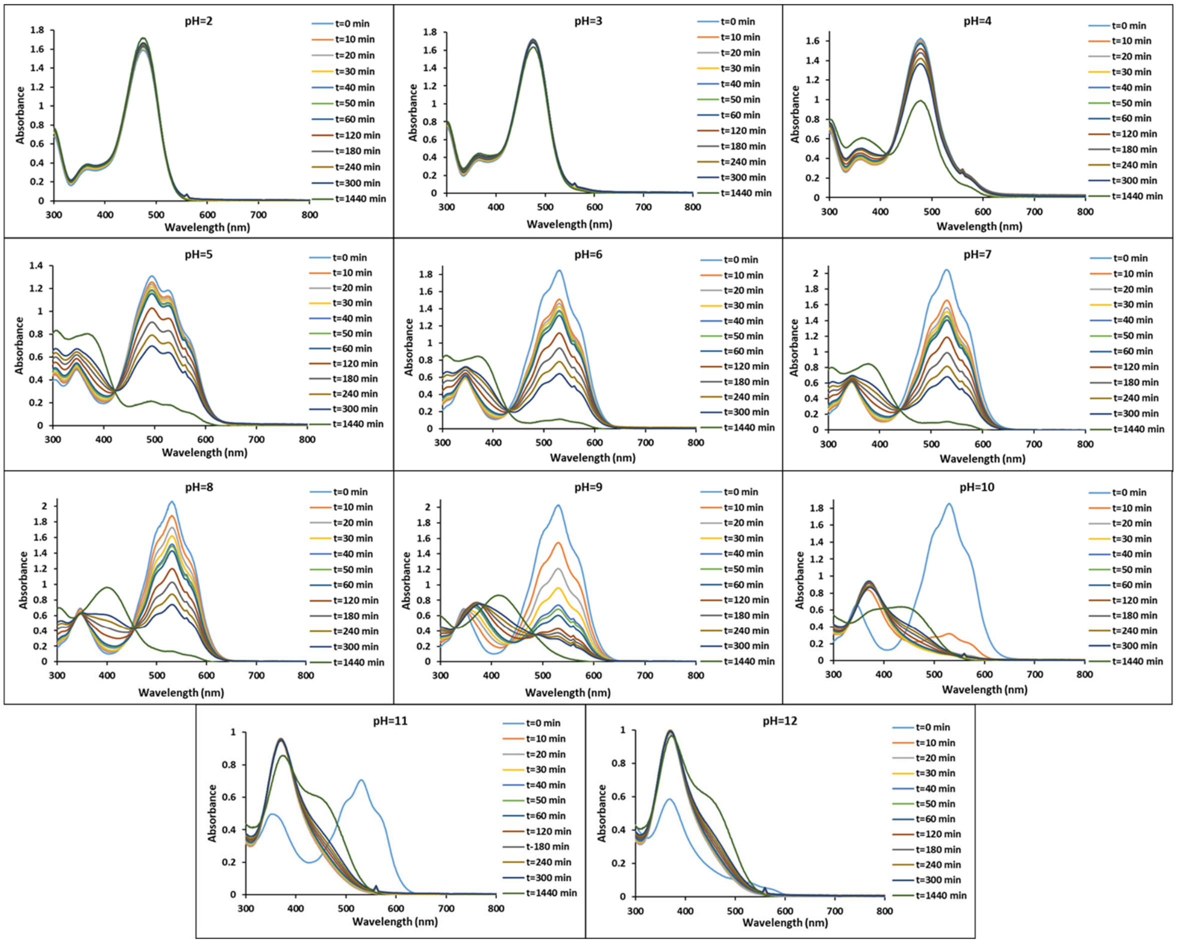

Figure S25. UV-Vis spectra of compound **5** solutions ($7{\cdot}10^{-5}$ M in methanol:water 1:14) at pH values ranging from 2 to 12

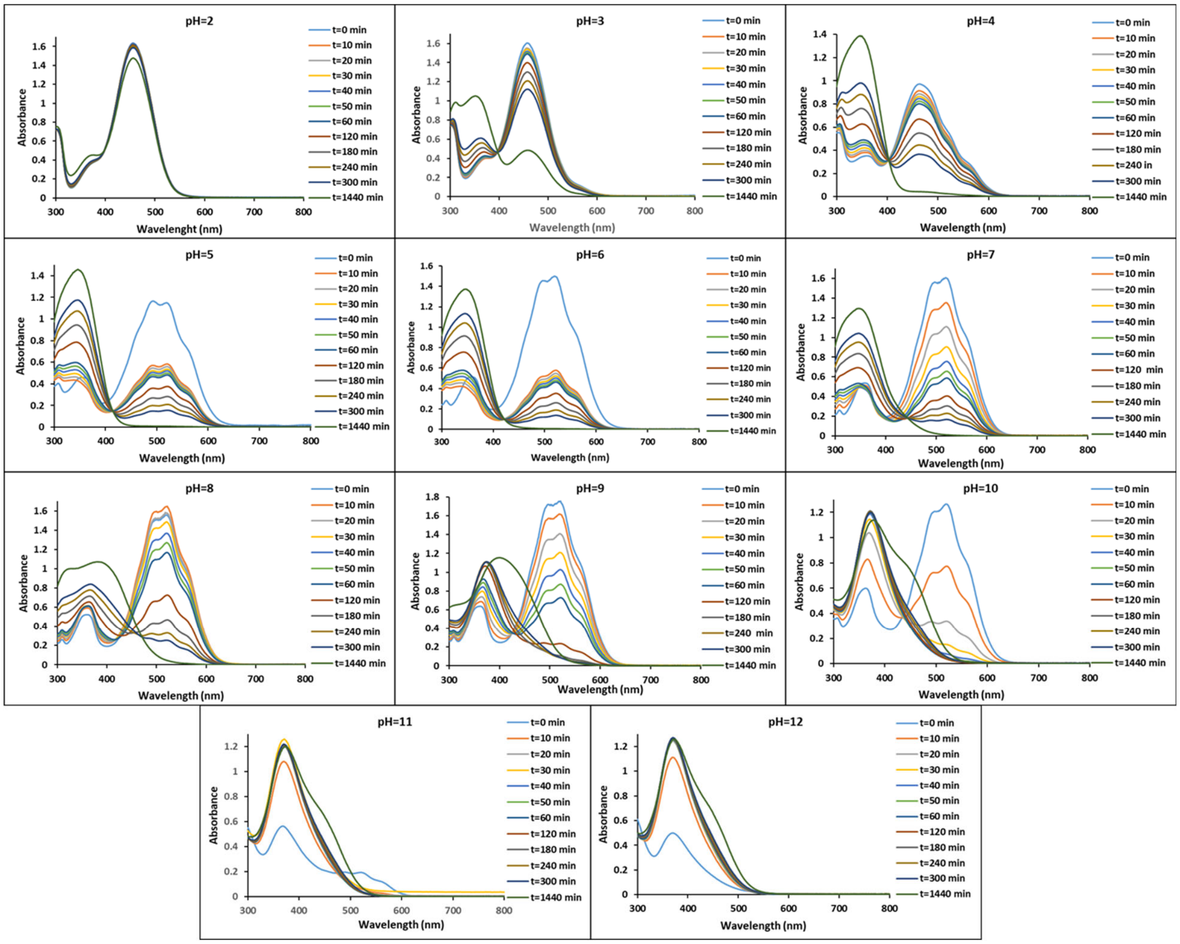

Figure S26. UV-Vis spectra of compound **7** solutions ($7{\cdot}10^{-5}$ M in methanol:water 1:14) at pH values ranging from 2 to 12

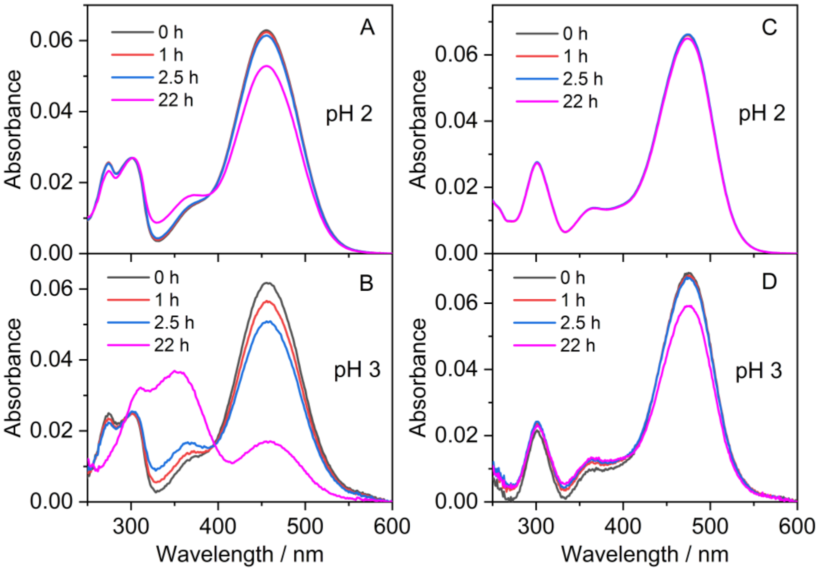

Figure S27. Absorption spectra for **7** at pH 2 (A) and pH 3 (B) as well as for **5** at pH 2 (C) and pH 3 (D) recorded at 0, 1, 2.5 and 22 h after preparation of the aqueous solutions.

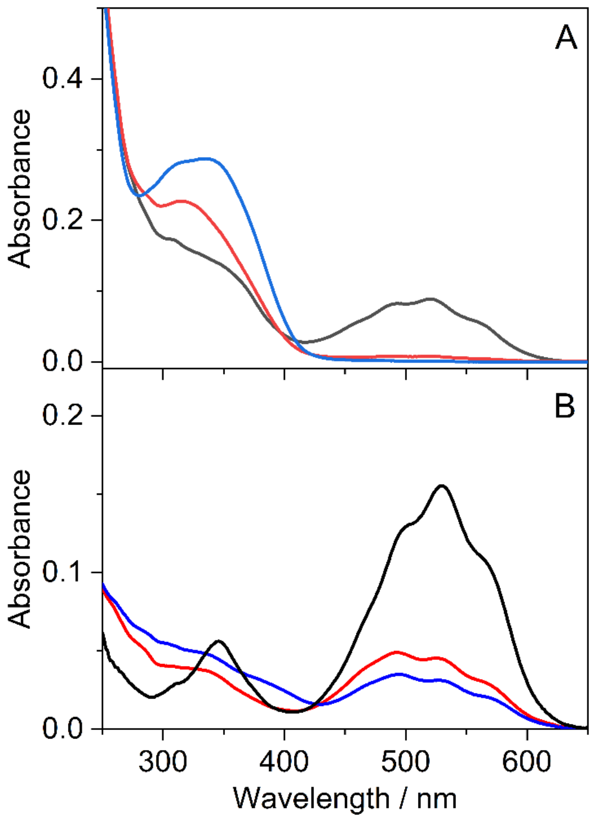

Figure S28. Absorption spectra of **7** (A) and **5** (B) immediately after the preparation of the samples in water (black lines) and in 11.43 mg/mL randomly methylated β-cyclodextrin aqueous solution (red lines). Blue lines display the spectra of the latter solutions in 3 h.

Table S1. t-test / Welch’s t-test / Mann-Whitney U-test performed for each concentration groups (C1 – 132 μM, C2 – 66 μM) in both cell lines.

Cell line	Sample	Normality test (Shapiro Wilk)]	*F*-test	*t*-test / Welch`s t-test / Mann-Whitney U test
HCT116	1	C1: $p = 0.326$<br>C2: $p = 0.685$	$F = 0.44$, num df = 7, denom df = 7, $p = 0.295$	$t = -3.21$, df = 14, $p < 0.01$**
	2	C1: $p = 0.331$<br>C2: $p = 0.989$	$F = 0.88$, num df = 7, denom df = 6, $p = 0.864$	$t = -5.64$, df = 13, $p < 0.001$***
	3	C1: $p = 0.223$<br>C2: $p = 0.484$	$F = 0.20$, num df = 7, denom df = 7, $p = 0.056$	$t = -24.1$, df = 13, $p < 0.001$***
	4	C1: $p = 0.771$<br>C2: $p = 0.969$	$F = 0.39$, num df = 7, denom df = 6, $p = 0.242$	$t = -4.36$, df = 13, $p < 0.001$***
HepG2	1	C1: $p = 0.356$<br>C2: $p = 0.999$	$F = 88.3$, num df = 7, denom df = 7, $p < 0.001$***	$t = -7.81$, df = 7.16, $p < 0.001$***
	2	C1: $p = 0.446$<br>C2: $p = 0.067$	$F = 4.32$, num df = 7, denom df = 7, $p = 0.072$	$t = 9.55$, df = 14, $p < 0.001$***
	3	C1: $p = 0.696$<br>C2: $p = 0.386$	$F = 0.25$, num df = 7, denom df = 7, $p = 0.091$	$t = -24.1$, df = 14, $p < 0.001$***
	4	C1: $p = 0.627$<br>C2: $p = 0.442$	$F = 2.81$, num df = 7, denom df = 7, $p = 0.196$	$t = 3.14$, df = 14, $p < 0.01$**

Table S2. One-way-ANOVA / Kruskal-Wallis test performed for each concentration groups (C1 – 132 μM, C2 – 66 μM, C3 – 26 μM) in both cell lines.

Cell line	Sample	Normality test (Shapiro Wilk)]	Homoscedasticity (Bartlett test)	one-way-ANOVA / Kruskal-Wallis	post-hoc (Tukey HSD / Nemenyi or Dunn)
HCT116	5	C1: $p = 0.266$<br>C2: $p = 0.275$<br>C3: $p = 0.663$	$p < 0.05$*	Chi-sq = 5.12, df = 2<br>$p > 0.05$	no differences
	6	C1: $p = 0.171$<br>C2: $p = 0.409$<br>C3: $p = 0.373$	$p = 0.241$	F = 43.40, df = 2<br>$p < 0.001$***	C1-C2: $p < 0.001$***<br>C1-C3: $p < 0.001$***
	7	C1: $p = 0.566$<br>C2: $p = 0.196$<br>C3: $p = 0.119$	$p < 0.001$***	Chi-sq = 14.68, df = 2<br>$p < 0.01$**	C1-C2: $p < 0.01$**<br>C1-C3: $p < 0.01$**
	8	C1: $p = 0.074$<br>C2: $p = 0.084$<br>C3: $p = 0.689$	$p < 0.05$*	Chi-sq = 15.52, df = 2<br>$p < 0.001$***	C1-C3: $p < 0.001$***<br>C2-C3: $p < 0.01$**
HepG2	5	C1: $p = 0.753$<br>C2: $p = 0.024$<br>C3: $p = 0.866$	$p < 0.05$*	Chi-sq = 14.65, df = 2<br>$p < 0.001$***	C1-C2: $p < 0.01$**<br>C1-C3: $p < 0.01$**
	6	C1: $p = 0.342$<br>C2: $p = 0.624$<br>C3: $p = 0.110$	$p < 0.001$***	Chi-sq = 19.60, df = 2<br>$p < 0.001$***	C1-C3: $p < 0.001$***<br>C2-C3: $p < 0.05$*
	7	C1: $p = 0.272$<br>C2: $p = 0.947$<br>C3: $p = 0.959$	$p < 0.001$***	Chi-sq = 18.61, df = 2<br>$p < 0.001$***	C1-C3: $p < 0.001$***<br>C2-C3: $p < 0.05$*
	8	C1: $p = 0.560$<br>C2: $p = 0.858$<br>C3: $p = 0.887$	$p = 0.993$	F = 346.1, df = 2<br>$p < 0.001$***	C1-C2: $p < 0.001$***<br>C1-C3: $p < 0.001$***<br>C2-C3: $p < 0.001$***

Table S3. One-way-ANOVA / Kruskal-Wallis test performed for each concentration groups (C1 – 132 μM, C2 – 66 μM, C3 – 26 μM) in the complexed samples on HepG2 cells.

Sample	Normality test (Shapiro Wilk)]	Homoscedasticity (Bartlett test)	one-way-ANOVA / Kruskal-Wallis	post-hoc (Tukey HSD / Nemenyi or Dunn)
5+SBECD	C1: p = 0.956<br>C2: p = 0.709<br>C3: p = 0.967	p < 0.001***	Chi-sq = 19.23, df = 2, p < 0.001***	C1-C3: p < 0.001***
7+RAMEB	C1: p = 0.342<br>C2: p = 0.386<br>C3: p = 0.246	p = 0.193	F = 14.44, df = 2, p < 0.001***	C1-C3: p < 0.001***<br>C2-C3: p < 0.05*

Table S4. t-test / Welch’s t-test / Mann-Whitney U-test performed for each concentration groups (C1 – 132 μM, C2 – 66 μM, C3 – 26 μM) to compare anthocyanidins (5, 7) with complexed ones (5+SBECD, 7+RAMEB) on HepG2 cell line.

Concentration group	Samples	Normality test (Shapiro Wilk)]	*F*-test	*t*-test / Welch`s t-test / Mann-Whitney U test
C1	5 – 5+SBECD	5: p = 0.753<br>5+SBECD: p = 0.956	F = 1.17, num df = 7, denom df = 6, p = 0.861	*t* = 3.84, df = 13, p < 0.01**
C2	5 – 5+SBECD	5: p = 0.240<br>5+SBECD: p = 0.709	F = 0.98, num df = 7, denom df = 7, p = 0.982	*t* = 3.81, df = 14, p < 0.01**
C3	5 – 5+SBECD	5: p = 0.866<br>5+SBECD: p = 0.967	F = 0.05, num df = 6, denom df = 7, p < 0.01**	*t* = -3.97, df = 7.80, p < 0.01**
C1	7 – 7+RAMEB	7: p < 0.01**<br>7+RAMEB: p = 0.674	F = 0.07, num df = 7, denom df = 6, p < 0.01**	W = 0, p < 0.01**
C2	7 – 7+RAMEB	7: p = 0.947<br>7+RAMEB: p = 0.992	F = 0.60, num df = 7, denom df = 6, p = 0.523	*t* = -11.3, df = 13, p < 0.001***
C3	7 – 7+RAMEB	7: p = 0.960<br>7+RAMEB: p = 0.246	F = 0.47, num df = 7, denom df = 7, p = 0.340	*t* = -11.2, df = 14, p < 0.001***
